# Mycobacterial IHF is a highly dynamic nucleoid-associated protein that assists HupB in organizing chromatin

**DOI:** 10.3389/fmicb.2023.1146406

**Published:** 2023-03-07

**Authors:** Joanna Hołówka, Tomasz Łebkowski, Helge Feddersen, Giacomo Giacomelli, Karolina Drużka, Łukasz Makowski, Damian Trojanowski, Natalia Broda, Marc Bramkamp, Jolanta Zakrzewska-Czerwińska

**Affiliations:** ^1^Department of Molecular Microbiology, University of Wrocław, Wrocław, Poland; ^2^Institute for General Microbiology, Christian-Albrechts-University, Kiel, Germany

**Keywords:** bacterial chromosome compaction, nucleoid-associated protein, mycobacteria, mycobacterial IHF, HupB

## Abstract

Nucleoid-associated proteins (NAPs) crucially contribute to organizing bacterial chromatin and regulating gene expression. Among the most highly expressed NAPs are the HU and integration host factor (IHF) proteins, whose functional homologues, HupB and mycobacterial integration host factor (mIHF), are found in mycobacteria. Despite their importance for the pathogenicity and/or survival of tubercle bacilli, the role of these proteins in mycobacterial chromosome organization remains unknown. Here, we used various approaches, including super-resolution microscopy, to perform a comprehensive analysis of the roles of HupB and mIHF in chromosome organization. We report that HupB is a structural agent that maintains chromosome integrity on a local scale, and that the lack of this protein alters chromosome morphology. In contrast, mIHF is a highly dynamic protein that binds DNA only transiently, exhibits susceptibility to the chromosomal DNA topology changes and whose depletion leads to the growth arrest of tubercle bacilli. Additionally, we have shown that depletion of *Mycobacterium smegmatis* integration host factor (msIHF) leads to chromosome shrinkage and replication inhibition.

## Introduction

Bacterial chromatin is a highly organized and yet dynamic entity. Due to the ongoing cellular processes, which are not spatiotemporally separated from one another, particular chromosomal regions must be accessible for the protein machineries involved in DNA-based processes, such as replication, segregation, and transcription. The bacterial chromosome is organized hierarchically, and there are many physical and biochemical factors keeping the order of its complex and dynamic architecture (Valens et al., 2004; Espeli et al., 2008; Dame et al., 2011, 2020; Badrinarayanan et al., 2015; Jeon et al., 2017; Lioy et al., 2018; Verma et al., 2019; Wasim et al., 2021). Several groups of relatively well-characterized factors are found in all bacteria and are responsible for either maintaining the topological homeostasis of the chromosomal DNA (i.e., topoisomerases) or its proper compaction (i.e., the SMC/MukB/Mks condensins and nucleoid-associated proteins, NAPs; Luijsterburg et al., 2006; Dame et al., 2011; Petrushenko et al., 2011; Wang et al., 2014; Marbouty et al., 2015). At present, little is known about how all of these factors combine to create the hierarchical structure of a bacterial chromosome. A particularly interesting model for such studies is genus *Mycobacterium*, which belongs to the Actinobacteria [currently named *Actinomycetota* (Oren and Garrity, 2021)]. The mycobacterial chromosome is known to be located asymmetrically within the cell (Hołówka et al., 2018), but its architecture is still unknown. Some studies have characterized proteins involved in chromosome organization, such as the NAPs and SMC condensins (Ghatak et al., 2011; Bhowmick et al., 2014; Panas et al., 2014; Hołówka et al., 2017; Odermatt et al., 2018; Kołodziej et al., 2021b), but it remains unclear how those elements cooperate to create the hierarchical and dynamic mycobacterial chromatin. Recently, we showed that the *Mycobacterium smegmatis* chromosome possesses a very characteristic structure (Hołówka et al., 2017, 2018). Using HupB (the *E. coli* HU protein homologue) fused with fluorescent protein (FP) as a chromosomal marker, we showed that the *M. smegmatis* chromosome possesses a unique bead-like structure wherein the beads exhibit dynamic behavior, constantly splitting and merging; however, the nucleoid generally occupies a similar amount of cellular space throughout the cell cycle (approx. 80% of the cell length in the exponential growth phase; Hołówka et al., 2017, 2018). Additionally, the mycobacterial chromosome (similar to those of other Actinobacteria) possesses a very high GC content, and nonpathogenic species have relatively large chromosomes (e.g., that of *M. smegmatis* is approx. 7 Mbp), which could also contribute to a unique chromosome morphology.

*Mycobacterium* possesses a unique set of NAPs, including the previously mentioned HupB protein and mycobacterial integration host factor (mIHF) (Supplementary Figures S1A,B), which is the *E. coli* IHF functional homologue (Pedulla and Hatfull, 1998; Prieto et al., 2012; Odermatt et al., 2018). HupB comprises an N-terminal domain whose 3D folding resembles that of other HU proteins (e.g., *E. coli* HU and *B. stearothermophilus* HU; Vis et al., 1995; Swinger and Rice, 2007; Bhowmick et al., 2014), and a C-terminal domain that is found exclusively in Actinobacteria and possesses several basic repeats characteristic of eukaryotic H1/H5 linker histones (Kumar et al., 2010; Gupta et al., 2014; Ghosh et al., 2016). The mIHF is known to be involved in phage integration (Pedulla and Hatfull, 1998), but shows no sequence or tertiary structure homology to its enterobacterial counterparts, which include the well-characterized IHF from *E. coli*. The tertiary structure of *E. coli* IHF is similar to that of HU, and both proteins belong to the same protein family. The mIHF resembles its homologue from an organism also belonging to Actinobacteria, *Streptomyces coelicolor* IHF (sIHF; approx. 70% sequence identity; Supplementary Figures S1C,E; Swiercz et al., 2013; Nanji et al., 2019). Besides completely different tertiary structure in comparison to other IHF proteins, *S. coelicolor* IHF (sIHF) was shown to bind DNA as a monomer, which also distinguishes it from HU and IHF (Swiercz et al., 2013; Nanji et al., 2019). These differences raised the question of whether mycobacterial IHF (mIHF) may have cellular functions analogous to those of *E. coli* IHF, such as contributing to the basic cellular processes (e.g., replication initiation; Ryan et al., 2002). Similar to *E. coli* IHF, mIHF was shown to be a DNA-bending protein that can also create rigid filaments and introduce left-hand loops, as assessed by Atomic Force Microscopy (AFM; Mishra et al., 2013; Odermatt et al., 2020a). Meanwhile, RNA-seq experiments revealed that *M. tuberculosis* IHF (mtIHF) influences the expression levels of many genes, including those involved in virulence (Odermatt et al., 2018). Both HupB and mtIHF bind DNA with no sequence specificity but exhibit preferences towards AT-rich sequences (Kumar et al., 2010; Hołówka et al., 2017; Odermatt et al., 2018). HupB was further shown to bind specific DNA structures, such as replication forks, nicked DNA, and ssDNA (Kumar et al., 2010; Sharadamma et al., 2011; Bhowmick et al., 2014). Our previous studies showed that the binding sites for HupB exhibited a bias against the *ter* region (most HupB-binding sites were located in the vicinity of *oriC*), prompting us to speculate that this protein may organize newly replicated *oriC* proximal regions (Hołówka et al., 2017). As seen for *E. coli* HU (Ryan et al., 2002), HupB is expected to be involved in forming the pre-replication complex. Interestingly, HupB is crucial only for pathogens (Bhowmick et al., 2014; Gupta et al., 2014), whereas mIHF is essential for the survival of both pathogenic and saprophytic bacteria (Odermatt et al., 2018).

To date, the studies on mIHF have been largely limited to *in vitro* analyses of mIHF-DNA interactions (Pedulla and Hatfull, 1998; Mishra et al., 2013; Sharadamma et al., 2015; Odermatt et al., 2020a). In the present study, we sought to explore the biological function of mIHF at the single-cell level and to understand its role in the organization of the mycobacterial chromosome alongside with HupB.

## Materials and methods

### DNA manipulations, bacterial strains, and culture conditions

The plasmids used to construct the *M. smegmatis* mc^2^ 155 strains were propagated in the *E. coli* DH5α strain. *Escherichia coli* cells were grown in LB broth or on LB agar plates (Difco) supplemented with the proper antibiotic(s) (ampicillin at 100 μg/ml, kanamycin at 50 μg/ml) and/or other compounds (5-bromo-4-chloro-3-indolyl-β-d-galactopyranoside [X-Gal] at 0.004%, isopropyl-β-d-1-thiogalactopyranoside [IPTG] at 0.5 mM). DNA manipulations were carried out using standard procedures (Sambrook et al., 1989). Reagents and enzymes were obtained from Thermo Scientific, Roth, and Merck. Oligonucleotides were synthesized by Merck, and sequencing was performed by Microsynth. *M. smegmatis* mc^2^ 155 strains were grown either in 7H9 broth supplemented with 10% ADC (albumin-dextrose-catalase; BD) and 0.05% Tween 80 or on 7H10 agar plates (Difco) supplemented with 10% OADC, 0.5% glycerol, 0.004% X-Gal, and/or kanamycin (50 μg/ml) or 2% sucrose. The construction of the *M. smegmatis* mc^2^ 155 strains is described in Supplementary Text S1, and all utilized oligonucleotides are listed in Supplementary Table S1.

### Fluorescence microscopy

Snapshot imaging was performed using log-phase cells (OD_600_, 0.6–1.0) or stationary-phase cells (OD_600_ ≥ 3). *Mycobacterium smegmatis* cultures were grown overnight in liquid medium, centrifuged (6,000 rpm, 5 min), and smeared onto microscopic slides or agar pads (1% agarose in 7H9 poured into 1.0 × 1.0-cm GeneFrames; Thermo Fisher Scientific; De Jong et al., 2011). Samples were examined with a Zeiss Axio Imager Z1 or a Leica DM6 fluorescence microscope equipped with a 100 ×/1.4 Oil objective. Images were taken using EGFP (up to 200 ms exposure time, 55% intensity) and mCherry (up to 300 ms exposure time, 100% intensity) channels.

For Time Lapse Microfluidics Microscopy (TLMM), early log-phase *M. smegmatis* cultures (OD_600_, 0.2–0.5) were grown in liquid medium. Experiments were performed by culturing cells in liquid medium using an ONIX microfluidic system (Trojanowski et al., 2015, 2019). Cells loaded into the observation chamber were cultured in fresh 7H9 medium supplemented with 10% ADC and 0.05% Tween 80 for 6 h; where indicated, the cells were exposed to an inhibitor (50 μg/ml novobiocin or 80 μM amsacrine) for 6 h and then washed for 8 h. Images were recorded at 10 min intervals (35 ms exposure time, 10% transmittance on transmitted light channel, 35–50 ms exposure time, 32% of transmittance on EGFP channel, and 80–150 ms exposure time, 50% transmittance on mCherry channel) using a Delta Vision Elite inverted microscope equipped with a 100 ×/1.4 Oil objective.

Microscopic data were analyzed using the Fiji and R software packages (R Foundation for Statistical Computing, Austria)^1^, including the ggplot2 package (Wickham, 2009). Data exhibiting Gaussian distribution were analyzed with a two-sided parametric Student’s *t* test. In the case of non-normal distributions, the statistical significance of the differences in measured values was confirmed with the nonparametric two-sided Wilcoxon test with minimum confidence intervals of 0.995.

### Lattice SIM imaging

Lattice SIM Imaging was performed using an Elyra 7 (Zeiss) inverted microscope equipped with an sCMOS 4.2 CL HS camera and an alpha Plan-Apochromat 100 ×/1.46 Oil DIC M27 objective. Samples (log-phase cells) were prepared on agar pads (1% agarose in 7H9 poured into 1.0 × 1.0-cm GeneFrames). Cells were illuminated with 488 nm laser (100 mW; 0.2% intensity) for EGFP fusions or with 561 nm laser (100 mW; 0.08% intensity) for SYTO82 staining. Each Z-plane was illuminated in Lattice-SIM mode comprising 13 phases, and for each phase cells were imaged for 30 ms. Image reconstruction was performed with the ZEN 3.0 SR software (Zeiss) with standard parameters and 3D models of the obtained data were prepared utilizing the VolumeViewer plugin (Fiji).

### Single-particle tracking (SPT)

Cells were cultured to mid-log phase in rich medium (7H9 supplemented with 10% ADC and 0.05% Tween 80). Slides and coverslips were cleaned by overnight incubation in 1 M KOH, washed with milli-Q water, and dried with pressurized air. Immediately before imaging, cells were spread onto agar pads (1% agarose in 7H9 poured into 1.0 × 1.0 cm GeneFrames; Thermo Fisher Scientific) and covered with clean 18 × 18 0.17-mm coverslips. Imaging was carried out using a Zeiss Elyra 7 inverted microscope equipped with an sCMOS 4.2 CL HS camera and an alpha Plan-Apochromat 63 ×/1.46 Oil Korr M27 Var2 objective (laser lines: 50 mW, 405 nm; and 100 mW, 561 nm). The Z-axis was stabilized *via* the Definite Focus.2 system (Zeiss). The samples were pre-bleached and the images were recorded using a 5 ms exposure per frame (561 nm laser, 100% intensity for fusions with PAmCherry, 80% for Dendra2, and 40% for HaloTag stained with TMRdirect, 10,000 frames in total) with constant 405 nm excitation (0.02% intensity for msIHF-PAmCherry and HupB-Dendra2; 0.05% intensity for HupB-PAmCherry and 0.01% for msIHF-Dendra2) in TIRF mode (62°angle). The temperature was maintained at 37°C during the imaging.

For SPT analysis, spots were identified and merged into tracks using the TrackMate v6.0.1 plugin (Fiji; Tinevez et al., 2017). Spot identification was performed within a diameter of 0.315 μm with the sub-pixel localization and median filter activated, and the signal to noise threshold was set at 5. Track reconstruction was performed with a maximum linking distance of 0.5 μm for PAmCherry and HaloTag fusions and 0.3 μm for Dendra2 fusions, no frame gaps allowed, and only tracks consisting of more than four frames being accepted for further analysis. Comparison and statistical analyses (by default Levene variance test or Student’s *t*-test) of the resulting HupB and msIHF single molecules trajectories were performed in SMTracker 2.0 (Oviedo-Bocanegra et al., 2021). The data were subjected to dwell time, mean-squared displacement (MSD), and squared-displacement (SQD) analyses (Rösch et al., 2018; Kołodziej et al., 2021b; Giacomelli et al., 2022). Dwell time was determined for a confinement radius of 97 nm and fitted with two components. MSD was calculated for four time points (with the last time point excluded), and fitted to the linear equation. In SQD analysis, determination of diffusion coefficients (D) from jump distances data (JD) was performed using independent fitting for msIHF vs. HupB, and using simultaneous fitting to compare mobility of msIHF in the wild-type and Δ*hupB* genetic background.

### msIHF overexpression and purification

The *Mycobacterium smegmatis ihf* (*msihf*) gene was PCR amplified from chromosomal DNA with primers P-1 and P-2 (Supplementary Table S1). The obtained PCR products were cloned into an EcoRI- and XhoI-cut pET28N-Strep expression vector by SLIC cloning (Jeong et al., 2012). Strep-msIHF protein was produced in *E. coli* BL21(DE3) containing pETN-Strep_msihf. When the culture reached OD_600_ = 0.5, fusion protein synthesis was induced by the addition of 0.25 mM IPTG. The culture was then incubated for 3 h at 37°C. Bacteria were harvested by centrifugation (10 min, 5000 *g*, 4°C) and pellets were stored at –20°C. The thawed cells were suspended in 1x buffer W (IBA Lifesciences, Cat. No. 2-1000-025) with the addition of a protease inhibitor (Thermo Fisher Scientific, Cat. No. A32963). After a 30 min incubation on ice, the cells were disrupted by sonication (10 min of pulse cycles: 5 s on, 5 s off at 50% amplitude; VCX 130 Vibra-Cell Ultrasonic Processor) and centrifuged (30 min, 4°C, 30000 *g*). The sample was centrifuged, Strep-Tactin Sepharose (IBA Lifesciences, Cat. No. 2-1,201-002) was added to the supernatant, and the mixture was incubated overnight at 4°C under gentle continuous agitation. The slurry containing bound strep-tagged proteins was transferred to a column, which was washed with buffer W until the Bradford test gave negative results. Elution was carried out with buffer W supplemented with 5 mM desthiobiotin. The elution fractions were supplemented with glycerol (10% final concentration) and stored at −80°C until further analysis.

### Electrophoretic mobility shift assay experiments

Electrophoretic mobility shift assay (EMSA) experiments were carried out as described previously (Wolanski et al., 2011), with some modifications. The near-infrared labeled 491-bp (fragment of *oriC*), 479-bp (~ 70% GC region), and 490-bp (*attB* site) DNA fragments were amplified with appropriate primers (Supplementary Table S1). An increasing amount of msIHF protein (0, 0.08, 0.16, 0.35, 0.7, 1.4, and 2.8 μM) was incubated with 50 fmoles of DNA fragment and 25 ng of poly(dI-dC) for 15 min at 20°C in DNA-binding buffer (50 mM Tris–HCl. [pH 8], 150 mM NaCl, 10 mM magnesium acetate, 0.02% Tween-20, 5% [*v*/*v*] glycerol, 1.0 mg/ml bovine serum albumin [BSA]) in a final volume of 20 μl. The reaction products were subjected to 1% agarose electrophoresis in 1 × TBE buffer at 4°C for 16 h at low voltage (2–3 V/cm). Fluorescence signals were detected using Azure 600 Imaging system (Azure Biosystems).

### Bio-layer interferometry (BLI)

Binding of msIHF protein to the *M. smegmatis* oriC region, *attB* site and GC-rich DNA was analyzed using a ForteBio Octet K2 system and Streptavidin biosensors (SAX2; Pall ForteBio). The analysis of a protein affinity for the linear biotinylated DNA was assessed at 30°C in BA buffer (PBS buffer supplemented with 50 mM Tris–HCl [pH 7.5] with 150 mM NaCl, 0.1 mg/ml BSA, and 0.05% (*v*/*v*) Tween-20). The wells of a 96-well black plate were filled with 200 μl of sample (msIHF concentrations: 0, 0.25, 0.5, 1, 2, 4, 8 μM) and incubated for 5 min at 30°C to allow the system to equilibrate. Then, 270 ng of biotinylated DNA fragments containing the oriC region, *attB* site or GC-rich DNA were immobilized on the sensor for 300 s. Thereafter, the sensor was regenerated with BB buffer (BA buffer supplemented with 0.05% SDS) for 300 s and neutralized with buffer BA for 300 s. Each round of protein-DNA binding analysis consisted of 60 s of sensor washing, 480 s of protein association, 180 s of protein dissociation, 300 s of sensor regeneration, and 300 s of sensor neutralization. The results were plotted as BLI sensorgrams after subtraction of the background signal obtained from the control well. The experimental data were analyzed using the software provided by the manufacturer of the equipment.

## Results

### msIHF colocalizes with the chromosomal marker, HupB, throughout the *Mycobacterium smegmatis* cell cycle

Our previous RNA-seq analysis (Kołodziej et al., 2021a) identified *hupB* and *msihf* as NAP-encoding genes that are highly expressed in the exponentially growing *M. smegmatis*, and products of those genes, HupB and msIHF, are functional homologs of *E. coli* HU and IHF, respectively (Supplementary Figure S1A). In the present work, our RT-qPCR experiments further showed that *hupB* and *mihf* genes are expressed at similar high level in the exponential phase, and theirs transcripts levels decrease in the stationary-phase cells (Figure. S1B). In this regard, msIHF appears to differ from its *E. coli* counterpart, which is produced at the highest level during the transition to stationary phase. As mentioned earlier, mycobacterial IHF does not share sequence or tertiary structure homology with its *E. coli* functional homologue, but its sequence is well conserved within Actinobacteria (see Supplementary Figures. S1C,E). Some reports suggested that (Sharadamma et al., 2015, 2017), in contrast to *M. smegmatis* IHF (msIHF), *M. tuberculosis* (mtIHF) includes an additional N-terminal domain (85 amino acids); recently, however, this hypothesis was rejected based on the finding that the longer *mihf* gene sequence arose from an incorrect annotation of the transcription start (Mishra et al., 2013; Odermatt et al., 2018). Moreover, we confirmed expression of the shorter version of mtIHF by Western blotting comparing lysates from *M. smegmatis* strains producing either mtIHF-EGFP or msIHF-EGFP fusion proteins (expressed under native promoters; Supplementary Figure S1D).

**Figure 1:**
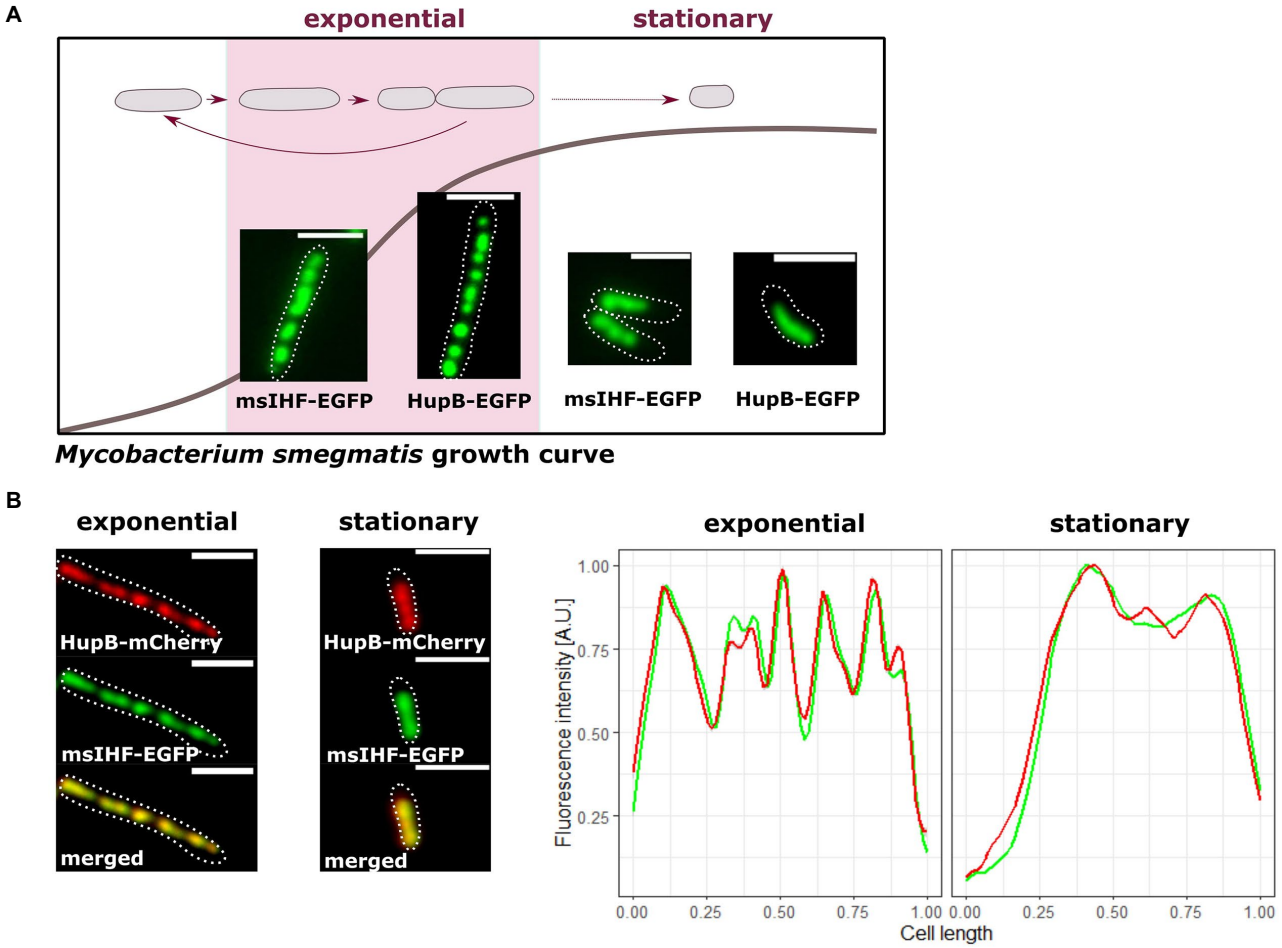
Comparison of the subcellular localizations of msIHF-FP and HupB-FP in *M. smegmatis*. **(A)** Representative cells of msIHF-enhanced green fluorescent protein (EGFP) and HupB-EGFP strains at the exponential and stationary phases of growth. **(B)** Colocalization of msIHF and HupB in *M. smegmatis* cells producing both proteins fused to EGFP and mCherry fluorescent proteins, respectively, instead of the native versions. Green and red fluorescence profiles collected from representative cells overlap, confirming the colocalization of the analyzed NAPs. Cell outlines are indicated with dotted white lines. Scale bar, 2 μm.

To explore the potential role of msIHF in chromosome organization, we first constructed several *M. smegmatis* fluorescent reporter strains and used them to elucidate the subcellular localization of msIHF. Since msIHF presumably binds chromosomal DNA as a monomer (Swiercz et al., 2013; Odermatt et al., 2020b), we utilized fusions with two different proteins: enhanced green fluorescent protein (EGFP) and mNeonGreen. Reporter strains exhibited growth (Supplementary Figure S2A) and colony morphology (data not shown) similar to those of the wild-type strain. Since *msihf* is an essential gene, these findings confirmed the functionality of the fusion proteins. Microscopic analyses revealed that both fusion proteins exhibited similar fluorescence patterns, and these patterns resembled those of HupB-EGFP-DNA macrocomplexes (see Figure 1A; Supplementary Figure S2B; Hołówka et al., 2017). However, the msIHF-FP foci were slightly larger and more dispersed in comparison to the distinctive and bright HupB-EGFP foci (Figure 1; Hołówka et al., 2017). msIHF-FP complexes were located along the long axis of the cell; it created a characteristic bead-like pattern and occupied an amount of intracellular space similar to that of HupB-EGFP complexes (Hołówka et al., 2017; approx. 75.0 ± 1.6% of the cell length in exponential phase, *n* = 150 and approx. 57.0 ± 0.4% in stationary phase, *n* = 150; Figure 1A). This observation suggested that msIHF may occupy the whole *M. smegmatis* chromosome, as seen for HupB (Hołówka et al., 2017). To confirm this, we constructed a strain producing HupB-mCherry and msIHF-EGFP instead of the corresponding native proteins (i.e., the *hupB* and *msihf* genes were exchanged for fusion genes in the native chromosomal loci; Figure 1B). Indeed, we observed colocalization of the red and green fluorescence patterns. Time Lapse Microfluidics Microscopy (TLMM) experiments showed that the msIHF-FP complexes exhibited a dynamic choreography during the cell cycle (see Movie 1) similar to that of HupB-FP, which we have previously characterized (Hołówka et al., 2017): The msIHF-EGFP complexes were splitting and merging. Prior to cell division, the foci separated into two clusters, indicating the occurrence of daughter chromosomes separation. The separation of the msIHF-EGFP complexes exhibited a timing comparable to that of the HupB-EGFP complexes (152 ± 24 vs. 155 ± 31 min, respectively; *n* = 100; *p* = 0.41).

Taking together, our results suggest that msIHF occupies the whole chromosome similarly to HupB.

### In contrast to HupB, msIHF particles are highly dynamic

**Figure 2:**
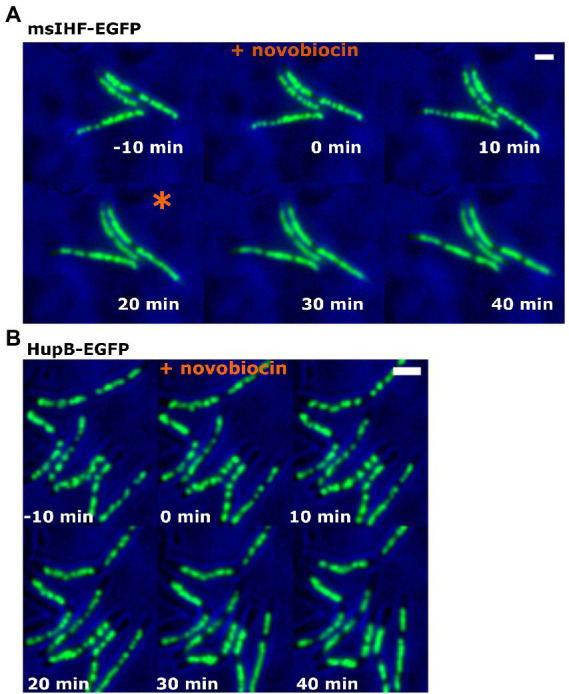
msIHF-EGFP and HupB-EGFP cells upon novobiocin treatment. *M. smegmatis* cells of msIHF-EGFP **(A)** and HupB-EGFP **(B)** strains were exposed to novobiocin (final concentration, 50 μg/ml) for 6 h. The moment at which the bead-like pattern disappeared in msIHF-EGFP strain cells is marked with an orange star. Scale bar, 2 μm.

The fluorescence patterns of the msIHF-FP-DNA and HupB-FP-DNA macrocomplexes were similar but their DNA-binding modes are known to vary significantly: In contrast to HupB, the msIHF homolog from *Streptomyces* was proposed to bind DNA as a monomer (Swiercz et al., 2013). Thus, we next compared the *in vivo* DNA-binding dynamics between the two NAPs. Since NAPs are the so-called “rapid reaction forces” of the cell, in that they mediate the cellular response to dynamically changing environmental conditions (Hołówka and Zakrzewska-Czerwińska, 2020), we analyzed the patterns of msIHF-EGFP and HupB-EGFP in cells exposed to stress (antibiotics treatment). Given that both msIHF and HupB seem to bind along the whole chromosome, we decided to use two antibiotics known to globally but oppositely affect chromosome structure/topology: Novobiocin is a gyrase inhibitor that causes DNA relaxation while amsacrine is a topoisomerase A inhibitor that increases DNA superhelicity. We utilized a previously described protocol (Szafran et al., 2018; Trojanowski et al., 2019) that applies TLMM to study the action of different antibiotics at a single-cell level. Microscopic analyses revealed that treatment with novobiocin, but not amsacrine, yielded some differences in the fluorescence patterns of msIHF-EGFP versus HupB-EGFP (see Figure 2; Movie 2). At 20 min after novobiocin was introduced into the observation chamber, all msIHF-EGFP cells lost their characteristic bead-like pattern (Figure 2A); in contrast, the HupB-EGFP complexes remained visible as distinct foci, but their sizes and distribution became more irregular than that seen before antibiotic treatment (Figure 2B); this effect was very heterogeneous and there was no obvious change in pattern, as seen for msIHF-EGFP (Figure 2A). These results suggested that increased chromosome relaxation (after novobiocin treatment) alters the binding of msIHF-EGFP to DNA and therefore the morphology of the msIHF-EGFP-DNA complexes (as evidenced by the loss of the bead-like pattern). Interestingly, after the antibiotic was washed away, many of the msIHF-EGFP cells recovered the bead-like pattern of their nucleoids (see Movie 2). Upon amsacrine treatment, in contrast, we did not observe any significant difference in the fluorescence patterns of msIHF-EGFP or HupB-EGFP (Supplementary Figure S3). Thus, it appears that the binding mode of msIHF, but not HupB, is altered upon topological change of the chromosomal DNA, but only in the case of increased chromosome relaxation. To further investigate the differences in the binding modes of msIHF and HupB *in vivo*, particularly in terms of their DNA-binding dynamics, we performed Single Molecule Localization Microscopy experiments (i.e., Single Particle Tracking, SPT).

**Figure 3:**
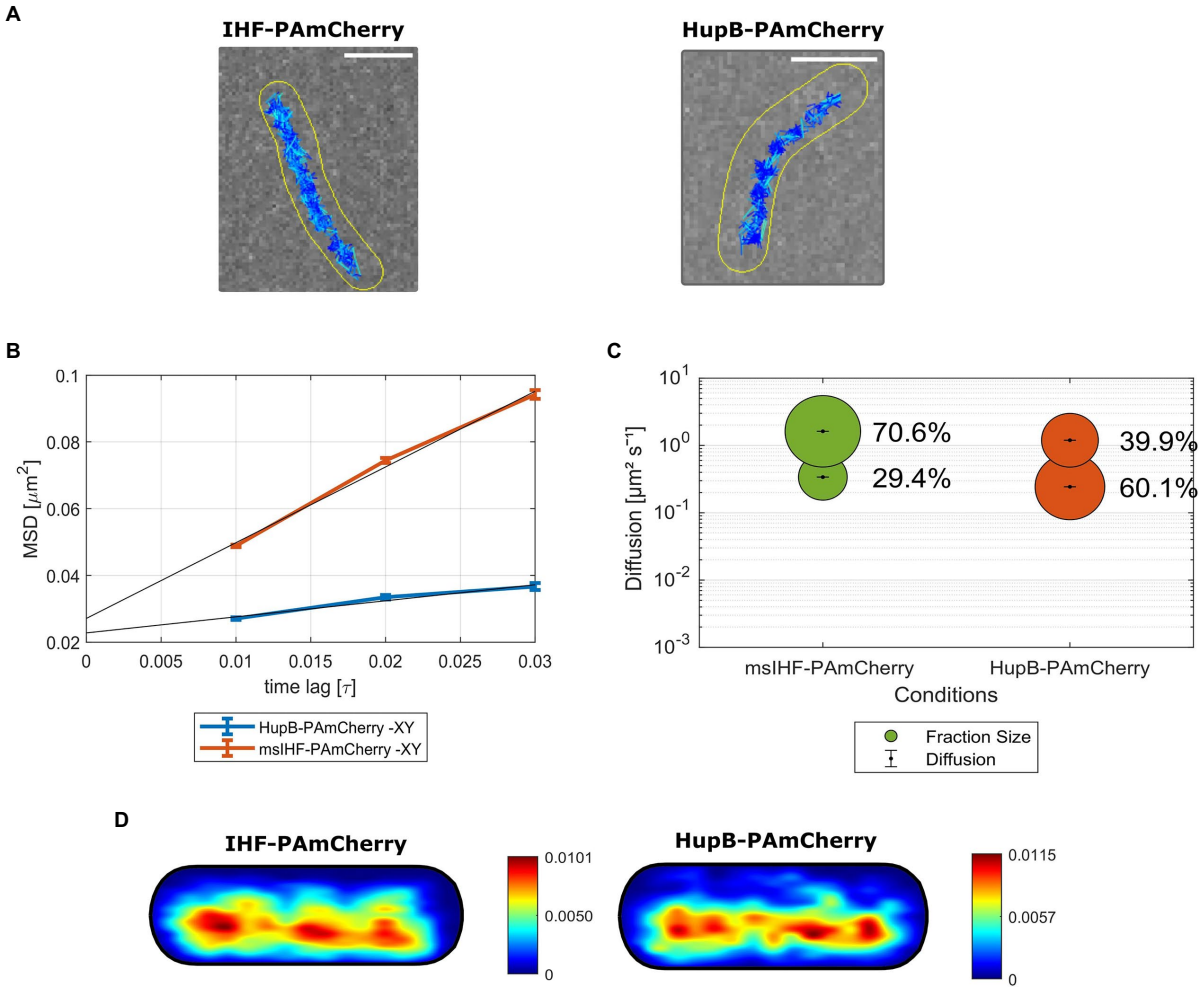
Single-particle tracking (SPT) analysis of msIHF-PAmCherry and HupB-PAmCherry particle mobility. **(A)** Representative cells of msIHF-PAmCherry and HupB-PAmCherry strains with identified tracks. The intensity of the dark blue color is inversely related to the length of the displacements within the presented tracks (i.e., the darkest tracks correspond to the trajectories of the most static molecules). Scale bar, 2 μm. **(B)** Mean-squared displacement plotted against time lag for the analyzed fusion proteins. **(C)** Bubble plot presenting obtained diffusion coefficients and the subpopulations sizes for msIHF-PAmCherry and HupB-PAmCherry molecules. **(D)** Heat maps for the average msIHF-PAmCherry and HupB-PAmCherry cells presenting likeness of presence of confined and mobile tracks from low (blue) to high (red).

To exclude the possibility that the mobility could be disturbed by the attached tags, we utilized several fusions for msIHF protein: the photoactivatable protein, PAmCherry; the photoconvertable protein, Dendra2; and the HaloTag enzyme, which binds fluorescently tagged ligands. In the case of HupB, we prepared fusions with PAmCherry and Dendra2. All constructed strains produced fusion proteins instead of the native versions of msIHF and HupB. Phenotypic analysis showed that fluorescent reporter strains producing PAmCherry and HaloTag fusion proteins exhibited growth rates similar to that of the wild-type strain, while the Dendra2 fusions caused a slight growth delay (Supplementary Figure S4A). Production of the fusion proteins was confirmed using microscopic analysis (for Dendra2 and HaloTag fusions; Supplementary Figure S4B) or Western blotting (for PAmCherry fusions; Supplementary Figure S4C). SPT analysis of single molecules of msIHF and HupB fused to PAmCherry (Figure 3) revealed that the particles of msIHF were much more dynamic than those of HupB. Identified trajectories for both msIHF-PAmCherry and HupB-PAmCherry particles were situated along the long cell axis (Figure 3A), but their mobilities differed significantly. Mean-squared displacement analysis showed that the average diffusion of msIHF-PAmCherry was almost 5 times higher than that of HupB-PAmCherry (diffusion coefficient, *D* = 0.56 μm^2^s^−1^ vs. *D* = 0.12 μm^2^s^−1^, respectively; Figure 3B). For proteins undergoing constant transitions, such as the DNA-binding events of msIHF and HupB, a more suitable method for estimating single-particles dynamics is jump distance (JD) analysis (see Supplementary Figure S4D). Squared displacement analysis (SQD, based on the JD of single molecules, independent fitting) corroborated the MSD results and further revealed that msIHF-PAmCherry molecules could be divided into two subpopulations: relatively confined particles (*D* = 0.338 ± 0.001 μm^2^s^−1^) and freely diffusive particles (*D* = 1.620 ± 0.001 μm^2^s−1), which constituted 29.4 and 70.6% of the analyzed particles, respectively (7,472 tracks from 68 cells; Figure 3C). In contrast, HupB-PAmCherry particles exhibited much lower mobility: The confined fraction constituted 60.1% (*D* = 0.243 ± 0 μm^2^s^−1^), while the diffusive fraction constituted only 39.9% (*D* = 1.190 ± 0.003 μm^2^s^−1^) of the analyzed molecules (5,368 tracks from 83 cells; Figure 3C). These differences in the population distributions were statistically significant (*p* < 0.001), preserved regardless of the exposure time, and the results obtained for HupB-PAmCherry corroborated our previous results showing that HupB possess a relatively high fraction of immobile particles (Hołówka et al., 2017). Population distributions for msIHF-Dendra2 was similar to msIHF-PAmCherry (74.4% of diffusive particles and 25.6% of confined particles), while the HupB-Dendra2 particles consisted of approx. Equal subpopulations (56 vs. 44% of diffusive and confined particles, respectively). However, in the case of Dendra2 fusions, the mobilities of both HupB-Dendra2 and msIHF-Dendra2 particles were lower (respectively *D* = 0.131 ± 0 μm^2^s^−1^ and *D* = 0.156 ± 0.001 μm^2^s^−1^ for HupB-Dendra2 and msIHF-Dendra2 confined particles, and *D* = 0.443 ± 0 μm^2^s^−1^ and *D* = 0.494 ± 0 μm^2^s^−1^ for HupB-Dendra2 and msIHF-Dendra2 diffusive particles) than those of the PAmCherry fusions (see above), seeming to suggest that fusion to Dendra2 somehow disturbed the mobilities of those proteins. Notably, there was also a slight growth delay of the strains producing Dendra2 fusion proteins (Supplementary Figure S4A). Dwell time analysis using a two-component model showed that there were 71.5% of msIHF-PAmCherry and 56.1% of HupB-PAmCherry particles resided in 97 nm radius for 0.040 s, and in the case of longer dwelling particles (i.e., 0.065 s) 28.5% of msIHF-PAmCherry and 43.9% HupB-PAmcherry, respectively. Hence, in contrast to HupB-PAmCherry, most of the msIHF particles seems not to remain bound to the DNA for long. Additionally, heat maps presenting the likelihood of confined molecules localization showed that confined molecules of both msIHF-PAmCherry and HupB-PAmCherry were distributed along the long cell axis in the area of the nucleoid (Figure 3D). We further confirmed the high mobility of msIHF molecules by analyzing micrographs of msIHF-EGFP and HupB-EGFP cells after cultures were fixed with 1% formaldehyde to freeze the DNA-molecule interactions (see Supplementary Figure S5). We did not observe any distinctive fluorescent foci in msIHF-EGFP as in the case of untreated control cells, whereas HupB-EGFP cells revealed the characteristic fluorescence pattern that represented HupB-EGFP-DNA complexes. We took this as indicating that the msIHF particles were changing their binding sites to quickly to “be caught” in specific places upon fixation.

Together, obtained results suggest that the DNA-binding modes of msIHF and HupB differ significantly: msIHF particles tend to be highly dynamic, and are susceptible to DNA topological changes (shown by the change of the msIHF-EGFP fluorescence pattern seen upon DNA relaxation); meanwhile, HupB exhibits a more stable binding mode regardless of the DNA topological status, and has a relatively large fraction of static particles compared to msIHF.

### Lack of HupB, a stable DNA-binding protein, alters chromosome morphology

**Figure 4:**
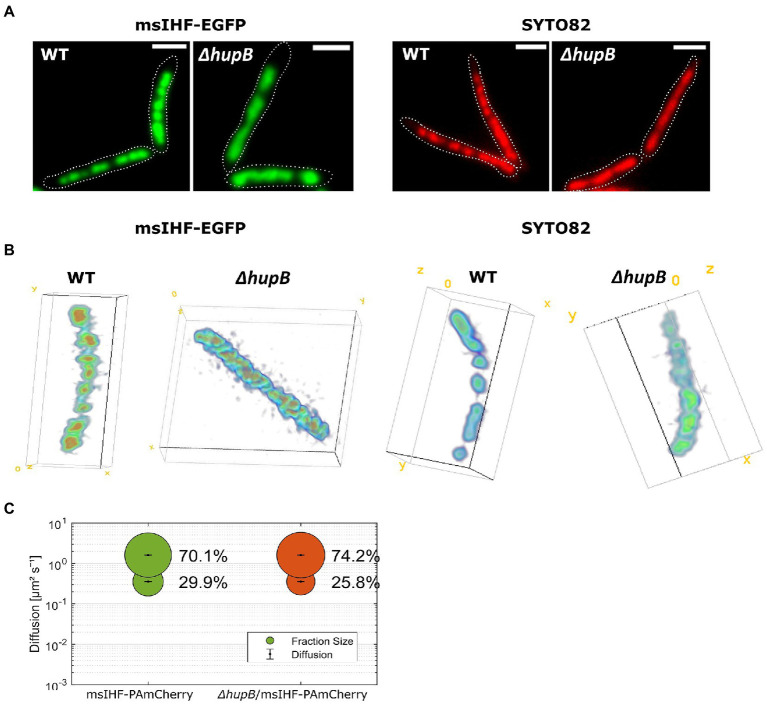
Analysis of changes in nucleoid morphology in msIHF-EGFP and *ΔhupB*/msIHF-EGFP strains in comparison to *ΔhupB* and wild-type *M. smegmatis* cells stained with SYTO82 chromosomal dye. **(A)** Wide-field epifluorescence microscopy-derived micrographs of representative cells from the analyzed strains. Cell outlines are indicated with dotted white lines. Scale bar, 2 μm. **(B)** Lattice SIM combined with Z-stacks of the analyzed strains. 3D models of the nucleoids were generated in Fiji using the Volume Viewer plugin. **(C)** Results of single-particle tracking experiments. Bubble plot presenting obtained diffusion coefficients and the subpopulations sizes for msIHF-PAmCherry and *ΔhupB*/msIHF-EGFP molecules.

We previously reported that the ∆*hupB* mutant strain exhibits a rather mild phenotype of delayed replication initiation (HupB is presumably involved in the pre-replication complex stabilization; Hołówka et al., 2018). Here, using msIHF-EGFP, we were able to investigate more closely the ∆*hupB* chromosome phenotype. Microscopic analysis of msIHF-EGFP strain deprived of HupB (∆*hupB*/msIHF-EGFP) showed that the characteristic bead-like pattern of the nucleoid was altered. In the deletion mutant, we did not observe the distinctive bright foci seen in the wild-type genetic background; instead, the fluorescence signals were more dispersed. This alteration in the fluorescence pattern was particularly visible in real-time experiments (see Movie 3; Figure 4A). Similar patterns were observed using the other fluorescent fusions of msIHF, namely those with mNeonGreen, Dendra2, and HaloTag (Supplementary Figure S6A). It is worth noting that we did not notice any significant change in *msihf* transcript level in the Δ*hupB* strain compared to the wild-type genetic background (Supplementary Figure S6B). To examine whether the observed nucleoid morphology alterations were simply the result of changes in the DNA-binding pattern of msIHF-FP, we stained the chromosome of ∆*hupB* strain cells with SYTO82 dye. Indeed, we observed a similarly dispersed signal lacking the distinctive foci of the wild-type strain, indicating that the chromosomal structure was altered in ∆*hupB* mutant cells (Figure 4A). This suggests that there is some change in the local chromosome structure of cells lacking HupB. Moreover, similar changes of the chromosome morphology we did observe in HupB depletion strain (see Supplementary Figure S7).

Next, we used Lattice SIM microscopy with Z-stacks to further investigate the chromosome structure of ∆*hupB*/msIHF-EGFP and ∆*hupB* strains at high-resolution and in 3D (Figure 4B). When using msIHF-EGFP fusion protein or chromosomal SYTO82 dye for visualization, we noticed similar alterations of the chromosome morphology in the ∆*hupB* genetic background: The wild-type nucleoid exhibited a characteristic bead-like structure, whereas the chromosome of the deletion mutant had a smoother rod-like shape (see Figure 4B).

To understand the nature of the changes observed in the chromosome architecture of ∆*hupB* strains, we performed SPT experiments using msIHF-PAmCherry fusion in ∆*hupB* strain (∆*hupB*/msIHF-PAmCherry; Figures S4A,C, S6C,D) in comparison to the wild-type genetic background. We hypothesize that, as a result of the structural alterations, we should be able to detect a change in msIHF single-particle mobility (Hołówka et al., 2017; e.g., caused by altered msIHF binding and/or dissociation) in the ∆*hupB* strain compared to the control strain similarly to the msIHF-EGFP pattern change seen upon novobiocin treatment of wild-type background cells (see Figure 2). However, our SPT experiments showed no obvious difference in msIHF particle mobility in the ∆*hupB* background compared to the wild-type background (see Supplementary Figure S6E). SQD analysis (simulations fitting for D determination) revealed that the subpopulations of dynamic molecules constituted 74.2 and 70.1% (*D* = 1.610 ± 0.002 $\mu m^{2}s^{-1}$ ) of the particles in ∆*hupB*/msIHF-PAmCherry and msIHF-PAmCherry, respectively, while confined particles comprised proportions of 25.8 and 29.9% (*D* = 0.356 ± 0.002 $\mu m^{2}s^{-1}$ ; Figure 4C), respectively. Interestingly, although both ∆*hupB*/msIHF-PAmCherry and msIHF-PAmCherry mostly comprised highly dynamic particles (74.2 vs. 70.1%; 6,916 tracks from 42 cells vs. 7,472 tracks from 68 cells, respectively), the between-strain difference was statistically significant (*p* = 0.003). Similar subpopulation distributions of msIHF particles in the *hupB* deletion mutant were observed when we used msIHF-Dendra2 (71.1 and 65.2% of diffusive particles, along with 28.9 and 34.8% of confined particles for Δ*hupB* and the wild-type genetic background, respectively) and msIHF-HaloTag fusion proteins stained with TMRdirect (70.4 and 64% of diffusive particles, along with 29.6 and 36% of confined particles for Δ*hupB* and the wild-type genetic background, respectively). Dwell time analysis revealed that there are more particles of msIHF-PAmCherry in the *hupB* deletion mutant dwelling for shorter time periods (83.6% for 0.040 s and 16.4% for 0.062 s) in comparison to the wild-type genetic background (71.5% for 0.04 s and 28.5% for 0.065 s), which is consistent with the higher mobility of msIHF-PAmCherry particles in the Δ*hupB* strain.

Next, we explored whether the altered chromosome morphology influences the phenotype on a single-cell level. As it was shown previously, population of ∆*hupB* cells exhibited only a slight growth delay at the population level (Supplementary Figure S6C; Hołówka et al., 2017). On the single-cell level, however, we noticed clear differences in cell length. In the exponential growth phase, deletion mutant cells were longer than wild-type cells (3.13 ± 0.98 vs. 2.93 ± 0.77 μm, respectively; *n* = 300, *p* = 0.006). A similar observation was made for ∆*hupB*/msIHF-EGFP versus msIHF-EGFP cells (4.54 ± 1.33 μm vs. 4.08 ± 1.09 μm, respectively; *n* = 300, *p* = $4\times 10^{-6}$ ). However, fusion protein-producing cells were generally longer than cells of the corresponding wild-type strains. The increase of cell length in cells lacking HupB may suggest that there is some delay in cell division, perhaps due to the prolonged segregation of morphologically altered chromosomes. To test this hypothesis, we performed TLMM and visualized the nucleoid with both msIHF-EGFP and SYTO82 chromosomal dye. Time-lapse imaging revealed that ∆*hupB* cells took longer than control cells to separate their daughter chromosomes (222 ± 52 vs. 194 ± 53 min, *n* = 120, *p* = $4.8\times 10^{-5}$ for ∆*hupB* and wild-type, respectively, under staining with SYTO82; 177 ± 30 vs. 161 ± 25 min, *n* = 80, *p* = 0.00058 for ∆*hupB*/msIHF-EGFP and msIHF-EGFP cells, respectively; Movies 1, 3). Interestingly, the ∆*hupB*/msIHF-EGFP strain seemed to be more fragile under blue light exposure than the msIHF-EGFP control strain. As we increased the exposure time and irradiation intensity for EGFP visualization, the deletion mutant exhibited a proportionally longer delay (up to 30 min; see Supplementary Table S2) of daughter chromosome separation. Previous reports showed that strains lacking HupB are more susceptible to UV radiation (Whiteford et al., 2011). Here, we show that they also seem to be more susceptible (in comparison to the control strain) to blue light (488 nm) exposure.

In summary, both conventional fluorescence microscopy and high-resolution experiments revealed that *M. smegmatis* strains lacking HupB protein exhibit altered nucleoid morphology. The change in the chromosome architecture of ∆*hupB* mutant strains was reflected in a delay of daughter chromosome separation. However, the lack of HupB had subtle effect on the dynamics of msIHF molecules, as demonstrated by SPT analysis.

### Depletion of msIHF leads to chromosome shrinkage and growth inhibition

Since *msihf* gene deletion is lethal (Pedulla and Hatfull, 1998; Odermatt et al., 2018), we utilized the CRISPRi/dCas9 system (Rock et al., 2017) to gradually silence the expression of this gene (for details see Text S1). We then observed how msIHF depletion affected chromosome dynamics and basic cellular processes, such as chromosome replication and segregation, in *M. smegmatis* cells. To allow us to easily monitor the decreasing protein levels, we constructed a fluorescence depletion strain (msIHF-EGFP↓) that produced msIHF-EGFP instead of the native protein. After dCas9 was induced with 50 ng/ml of anhydrotetracycline (aTc), the transcription of the *msihf-egfp* gene was blocked and the initial msIHF-EGFP protein population (that produced before dCas9 induction) was diluted in the subsequent generations of daughter cells (Supplementary Figure S8). Microscopic analysis showed that system worked effectively: The growth rate of msIHF-EGFP↓ strain was much slower under induction with aTc (Supplementary Figure S8A) and the fluorescence signal coming from the msIHF-EGFP fusion protein disappeared gradually as the generations progressed (see Supplementary Figure S8C). As the level of msIHF-EGFP dropped below a critical point, the cells stopped growing (after approx. 4–5 generations of cells; estimated from the TLMM experiments). However, we did not observed any msIHF-EGFP pattern alterations before the signal disappeared suggesting msIHF depletion does not affect chromosome morphology (Figure. S8C). The decreases in the mRNA and protein levels of msIHF in the msIHF-EGFP↓ strain were confirmed by RT-qPCR and Western blotting, respectively (Supplementary Figures S8B,D). To analyze the chromosome structure, chromosome replication dynamics, and segregation in the *msihf* depletion background, we constructed the following strains: HupB-FP/msIHF↓ (HupB fused with EGFP and mCherry; chromosome markers (Hołówka et al., 2017), DnaN-mCherry/msIHF↓ [replication marker (Trojanowski et al., 2015)], and ParB/msIHF↓ [chromosome segregation marker (Trojanowski et al., 2015)]. The depletion of msIHF resulted in growth delay of constructed mutant strains (Supplementary Figure S9A).

**Figure 5:**
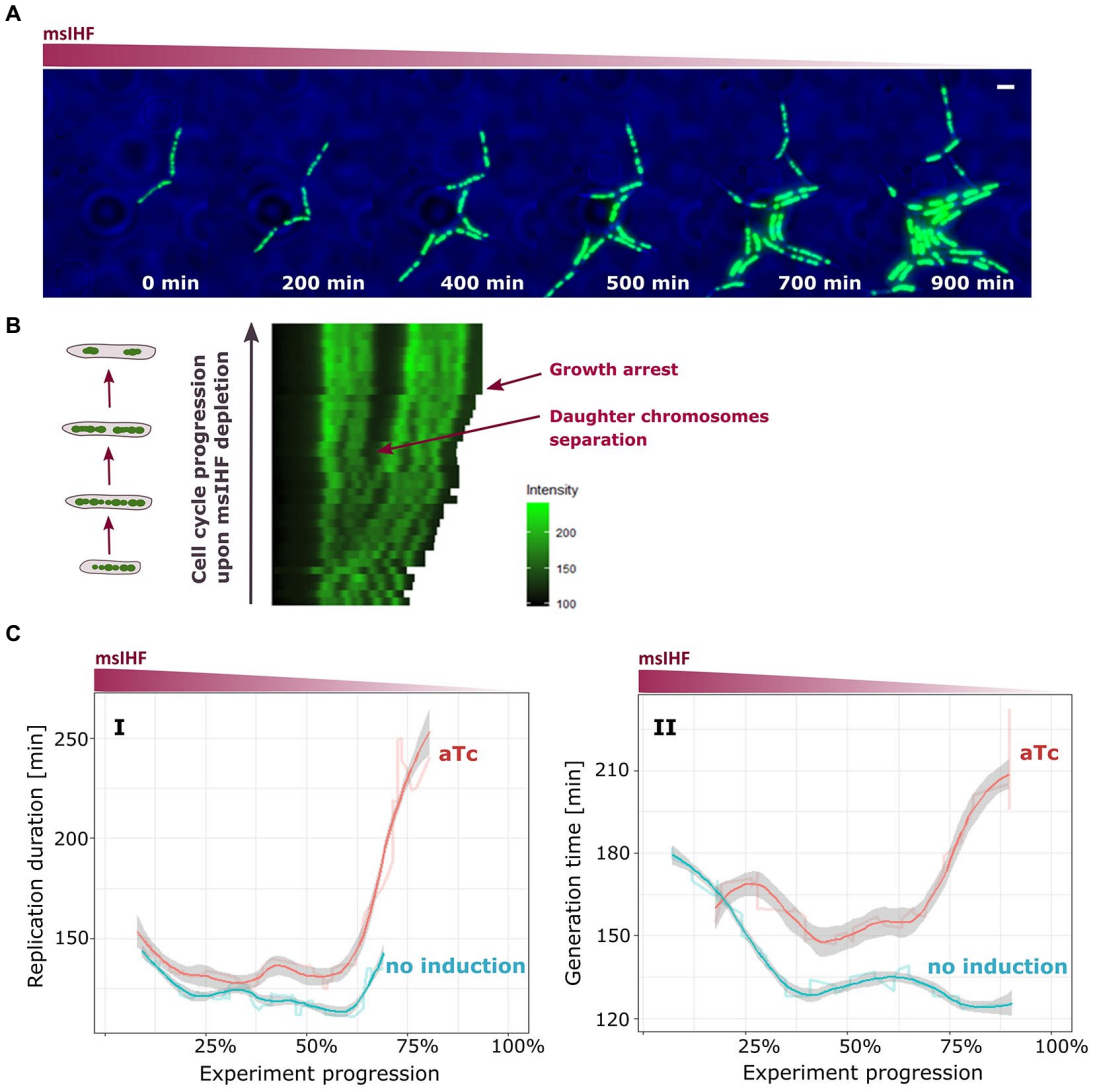
Influence of msIHF depletion on chromosome organization, replication, and segregation dynamics in *M. smegmatis*. **(A)** Time-lapse experiment of HupB-EGFP/IHF strain showing nucleoid shrinkage after induction of dCas9 with 50 ng/ml aTc. Scale bar, 2 μm. **(B)** Kymograph of representative cells of HupB-EGFP/msIHF↓ strain upon aTc induction. **(C)** Charts present average replication time (**I**; duration of chromosome replication) and generation time (**II**; time between two subsequent *oriC* doubling events) in DnaN-mCherry/msIHF↓ and ParB-mCherry/msIHF↓ strains during the progression of TLMM experiments (i.e., moving average). DnaN-mCherry/msIHF↓ and ParB-mCherry/msIHF↓ strains were induced with 50 ng/ml of aTc and compared to the corresponding strains grown without inductor (i.e., the native msIHF level).

Time Lapse Microfluidics Microscopy experiments showed that the mycobacterial chromosome (HupB-EGFP/msIHF↓ strain) started to shrink rapidly after approx. 4–5 generations post-dCas9 induction, until eventually the cells stopped growing (see Figures 5A,B). Similar results were obtained for HupB-mCherry/msIHF↓ (i.e., nucleoid shrinking after 4–5 cells generations). Interestingly, for the initial generations prior to chromosome shrinkage, the dynamics of daughter chromosome separation and chromosome morphology seemed to be unaffected (Figures 5A,B). Thus, it suggests that msIHF, in contrast to HupB, may not be crucial for maintaining chromosome architecture, and *Mycobacterium* needs only a minimal concentration of msIHF for growth. We also monitored the progression of replication and segregation in DnaN-mCherry/msIHF↓ and ParB-mCherry/msIHF↓ strains upon induction of Cas9, using TLMM. Similar to our observations for HupB-EGFP/msIHF↓, the cells seemed to be unaffected at the beginning of the experiment, when the msIHF concentration was presumably still sufficient. As expected, both replication and segregation were affected after approximately 4–5 generation of cells, when the available population of msIHF molecules was presumably too low: The durations of these processes eventually became lengthened (Figure 5C). When we examined the time between the termination of the previous round and initiation of the next round of replication (phase B + D) upon Cas9 induction, we found that the average time of phase B + D was also prolonged in comparison to the not induced control cells. As the experiment progressed, the average values of the B + D phase duration (Supplementary Figure S9B) became more varied than seen in our analyses of the replication (phase C of the cell cycle) and segregation dynamics analyses presumably because B + D phase is the most heterogeneous part of the mycobacterial cell cycle.

**Figure 6:**
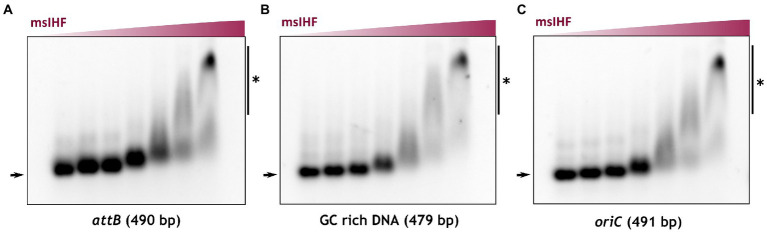
Analysis of msIHF DNA binding *in vitro*. Electrophoretic mobility shift assay (EMSA) results. Linear DNA fragments including the *attB* site **(A)** (positive control), *oriC* **(B)**, and GC-rich DNA **(C)** (negative control) were incubated with increasing concentrations of recombinant msIHF (0, 0.08, 0.16, 0.35, 0.7, 1.4, and 2.8 μM) and a nonspecific competitor (poly dI-dC). Nucleoprotein complexes created by the binding of msIHF with the analyzed DNA fragments are marked with vertical lines and a black star.

Our microscopic observations indicated that msIHF depletion had notable effects only when the level was significantly reduced. This suggested that only a small amount of msIHF is required for the cells to grow (Supplementary Figure S8). Thus, the function of msIHF in maintaining chromosome architecture is presumably not crucial for survival. We postulated that msIHF may be essential to other processes, such as through regulating the expression levels of certain genes and/or participating in some basic cellular processes, such as chromosome replication. Recent data showed that *M. tuberculosis* IHF (mtIHF) positively regulates the expression levels of *dnaA* and *dnaN*, which encode the DnaA initiator protein and the β-subunit of DNA polymerase III, respectively (Odermatt et al., 2018). Moreover, *E. coli* IHF protein enables the initiation of chromosome replication by binding within the *oriC* region and facilitating the formation of a DnaA filament (Ryan et al., 2002). Thus, we speculated that *M. smegmatis* IHF (msIHF) may be also involved in the replication initiation. To test this hypothesis, we analyzed msIHF-*oriC* interactions *in vitro* by performing electrophoretic mobility shift assay (EMSA) experiments. Since msIHF was originally identified as a factor required for mycobacteriophage L5 integration, but it does not bind the phage attachment site (*attP*) specifically like its *E. coli* homologue (Pedulla and Hatfull, 1998), we decided to test if msIHF binds a DNA fragment containing the bacterial attachment site (*attB*). Additionally, to exclude the non-specific binding of msIHF, we used a high-GC fragment (70% GC content) as a negative control. The results showed that msIHF bound with similar affinity to both *attB* site and *oriC* region from *M. smegmatis* (62% GC content; Figures 6A,B), and also to fragment containing the high-GC negative control (Figure 6C). To confirm those observations, we additionally utilized Bio-Layer Interferometry (BLI). Analysis of the affinity of msIHF towards a linear biotinylated DNA fragment encompassing *attB* site, *oriC* region or high-GC control confirmed the results of our EMSA experiments. The obtained BLI data (Supplementary Figure S10) showed that msIHF binds the *oriC* region with similar affinity as the two other fragments (K_D_*oriC*_ = 0.27 μM, K_D_*attB*_ = 0.25 μM and K_D_highGC_ = 0.27 μM). In each case we observed sharp sensorgram shapes at the beginnings of the protein association and dissociation steps, suggesting the presence of a dynamic protein-DNA interaction (see Supplementary Figure S10). Additionally, our data fitting and calculations excluded a cooperative binding of msIHF molecules. Those results are consistent with our SPT analysis showing that single msIHF particles exhibit very high mobility, which may be the reason we were not able to show msIHF specific binding of the *oriC* region *in vitro*.

In summary, our results indicate that msIHF is crucial for mycobacterial survival, and the dynamic behavior of this protein observed in super-resolution microscopic experiments was corroborated by *in vitro* studies of the msIHF binding mode.

## Discussion

**Figure 7:**
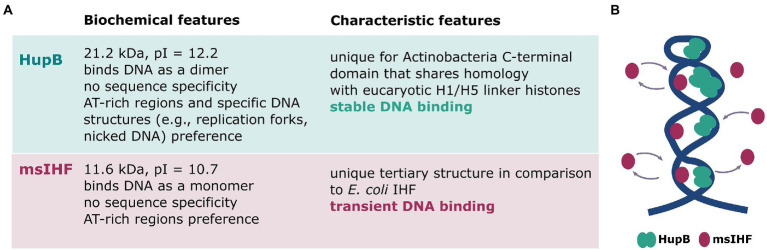
Characteristics of HupB and msIHF. **(A)** Biochemical characteristics of msIHF and HupB (Kumar et al., 2010; Bhowmick et al., 2014; Odermatt et al., 2018, 2020a). **(B)** Hypothetical model of the interplay of msIHF and HupB in local-scale chromosomal DNA binding. HupB molecules create a scaffold to maintain the local structure of the chromosomal DNA, and msIHF particles transiently bind the DNA.

Similar to eukaryotic chromatin, bacterial chromosomes are highly organized and form ordered, hierarchical structures (Dame et al., 2019, 2020). Our previous studies showed that a single *M. smegmatis* chromosome is located asymmetrically within the cell (closer to the new cell pole) and has a very distinctive morphology, wherein several dynamic beads split and merge during the cell cycle to create a characteristic bead-like structure (Hołówka et al., 2017, 2018). To better understand the complex architecture of the mycobacterial chromosome, we set out to explore the functions of two unique NAPs that are expressed at high levels in *M. smegmatis*: msIHF and HupB (for details see Figure 7A).

Microscopic observations revealed that, similar to HupB, msIHF complexes creates a characteristic bead-like pattern (see Figure 1A; Movie 1; Supplementary Figures S2, S4; Hołówka et al., 2017) and both proteins colocalize (Figure 1B). This strongly suggests that msIHF also occupies the whole chromosome. Recent data showed that mtIHF binding sites are distributed along the whole chromosome (ChIP-seq data; Odermatt et al., 2018). Notably, HU and IHF proteins that also bind along the whole *E. coli* chromosome (Wang et al., 2011), in contrast to HupB and mIHF, belong to the same protein family (Prieto et al., 2012). Furthermore, HU is produced mainly in actively dividing cells, whereas the level of IHF increases during the transition to the stationary phase (Dillon and Dorman, 2010). In the case of mycobacteria, both *hupB* and *msihf* are highly expressed during the exponential phase of growth (Supplementary Figures S1A,B). This suggests that, similarly to HupB, msIHF may contribute to organizing chromosomal DNA and regulation of certain cellular processes.

Despite similar localizations of both proteins, binding of msIHF, unlike that of HupB, appears to be sensitive to the lowered level of chromosomal DNA superhelicity (Figure 2; Movie 2) suggesting that msIHF may not bind chromosomal DNA as stably as HupB. Our further analysis using single-particle tracking (SPT; Figures 3A,D) corroborated this hypothesis: msIHF was found to be a much more dynamic protein than HupB, with the former comprising approximately 2.4 times more mobile particles than HupB (Figures 3B,C). We postulated that the highly dynamic behavior of msIHF particles could reflect transient DNA-binding events arising from the characteristic DNA binding mode of this protein, which presumably binds DNA with a low affinity as a monomer, in a manner similar to its *S. coelicolor* homolog (sIHF; Swiercz et al., 2013). Additionally, dynamic behavior of msIHF molecules was confirmed by analyzing micrographs of msIHF-EGFP and HupB-EGFP cells after fixation with formaldehyde where msIHF-EGFP, in contrast to HupB-EGFP, lost its characteristic fluorescence pattern (Supplementary Figure S5). Given this high mobility of msIHF particles, we speculate that this NAP assists HupB in chromosome organization on a local scale, and that its function may be crucial under certain environmental and/or intracellular stress conditions, such as changes of DNA topology.

Next, we sought to examine the chromosome morphology and dynamics in cells lacking HupB. Our microscopic experiments revealed that the lack of HupB (Figures 4A,B; Supplementary Figure S6A; Movie 3) or its decreased level (Supplementary Figure S7) altered the nucleoid morphology: Instead of discrete foci along the cell, the deletion mutant exhibited a more dispersed fluorescence pattern. This might reflect disturbed chromosome architecture presumably due to local-level chromosome decondensation, as such changes were previously observed in *E. coli* cells lacking HU protein (Macvanin et al., 2012) or observed in our experiments upon novobiocin treatment (see Figure 2; Movie 2). Moreover, we suggest that HupB protein ensures survival under stress factors, such as blue light exposure (Supplementary Table S2). Our observations showed that HupB plays a crucial role in maintaining local-scale chromosome architecture, which in turn allows the formation of higher chromosomal structures visible as the characteristic bead-like pattern in HupB-EGFP cells. Interestingly, the SPT experiments of msIHF particles in wild-type versus Δ*hupB* genetic background showed that there were only slight differences in the msIHF subpopulation distribution, and that both wild-type and deletion mutant strain cells had mostly highly mobile msIHF molecules (Figure 4C). These results suggest that the disturbed chromosome morphology in the cells lacking HupB may arise from a combination of alterations in local chromosome compaction and increased availability of the binding sites for msIHF particles. It is possible that a population of msIHF molecules persists in close proximity to the nucleoid and transiently binds chromosomal DNA in a constant state of dynamic exchange (Figure 7B). This type of binding mode might create a proficient system of DNA protection and/or gene regulation under stress conditions. Further studies would be required to confirm this hypothesis.

In addition to their structural role, NAPs are usually involved in various cellular processes, such as chromosomal DNA replication or transcription regulation (Ryan et al., 2002; Luijsterburg et al., 2006; Dillon and Dorman, 2010; Gordon et al., 2010; Flores-Ríos et al., 2019). Recent RNA-seq studies revealed that, as seen for *E. coli* IHF (Prieto et al., 2012), *M. tuberculosis* IHF (mtIHF) regulates the expression levels of certain housekeeping genes, including those involved in chromosome replication (Odermatt et al., 2018). Thus, we investigate the dynamics of basic cellular processes in cells with silenced *msihf* gene (Supplementary Figure S8). Microscopic analyses of msIHF depletion strains revealed that the cells exhibited rapid chromosomal shrinkage followed by growth inhibition after several generations (Figures 5A,B), similarly to cells exposed to the nalidixic acid (gyrase inhibitor; Trojanowski et al., 2019). Hence, we postulate that nucleoid shrinking in msIHF-depleted strains is a response to the severe stress condition. Our further experiments showed that, unlike the case of HupB, decreasing the level of msIHF did not affect the chromosome structure at the early stages of the cell cycle (Figure 5A; Supplementary Figure S8C). Moreover, the replication and segregation duration was prolonged only when the level of msIHF drops critically (see Figure 5C). Hence, our collective results suggest that relatively little amount of msIHF protein is required for the cells to survive. Given this, along with the observation that *msihf* deletion is lethal, we speculate that msIHF may be required for specific cellular processes that rely on the dynamic DNA transactions. Since, RNA-seq and ChIP-seq experiments (Odermatt et al., 2018) show that *M. tuberculosis* IHF positively regulates the expression levels of the *oriC* flanking genes *dnaA* and *dnaN*, it would be possible that msIHF may influence the replication process not only by regulating their expression but also, similar to its functional homolog, *E. coli* IHF (Ryan et al., 2002), by binding within *oriC* to facilitate proper pre-replisome complex formation. However, our *in vitro* studies of msIHF binding to the *oriC* region did not corroborate this hypothesis, and revealed that msIHF binding is not specific neither to *oriC* region, nor to *attB* site (Figures 6B,C; Supplementary Figure S10). These results are consistent with previous findings showing that the msIHF homolog from *Streptomyces* (sIHF) binds DNA as a monomer with no sequence specificity (Swiercz et al., 2013; Nanji et al., 2019), and with our microscopic analyses, suggesting msIHF is a highly mobile NAP, susceptible for DNA topological changes (Figures 2, 3). Hence, our findings do not exclude the involvement of msIHF in pre-replication complex formation or the mycobacteriophage L5 integration (Pedulla and Hatfull, 1998). Possibly, we were not able to reconstruct the *in vivo* conditions (i.e., proper level of superhelicity of the analyzed fragments) enabling a specific binding of msIHF. Additionally, it was reported that *E. coli* IHF exhibits multiple binding modes (Lin et al., 2012; Velmurugu et al., 2018; Yoshua et al., 2021); besides the site-specific binding, IHF can bind chromosomal DNA in non-specific manner, which is characteristic feature for NAPs.

We herein show that the msIHF-FP and HupB-FP proteins possess very similar localization within *M. smegmatis* cells but differ significantly in their DNA-binding modes *in vivo*. HupB maintains proper chromosome condensation/organization on a local scale and lack of it causes alterations in the chromosome architecture, while msIHF is a highly dynamic protein that binds only transiently to chromosomal DNA (see Figure 7B). It remains unclear why msIHF is produced on a level that enables it to occupy the whole chromosome, as does HupB. Possibly the dynamic, non-specific binding of msIHF could be required in certain conditions (e.g., under stress) to regulate gene expression and/or compact chromosomal DNA.

## Data availability statement

The original contributions presented in the study are included in the article/Supplementary material, further inquiries can be directed to the corresponding author.

## Author contributions

JH, JZ-C, DT, and MB contributed to the conception and design of the study. JH, HF, and GG optimize and performed super-resolution microscopy experiments. JH wrote the original draft, performed analysis, and visualized data. JH, TŁ, ŁM, KD, and NB performed experiments. JZ-C, TŁ, DT, and MB reviewed and edited the manuscript. JZ-C and MB delivered resources. JH and JZ-C raised funds. All authors contributed to the article and approved the submitted version.

## Funding

This work was financed by a National Science Centre, Sonatina 2 (2018/28/C/NZ1/00128), and Opus 19 (2020/37/B/NZ1/00556) grants.

## Conflict of interest

The authors declare that the research was conducted in the absence of any commercial or financial relationships that could be construed as a potential conflict of interest.

## Publisher’s note

All claims expressed in this article are solely those of the authors and do not necessarily represent those of their affiliated organizations, or those of the publisher, the editors and the reviewers. Any product that may be evaluated in this article, or claim that may be made by its manufacturer, is not guaranteed or endorsed by the publisher.

## Footnotes

^1^http://www.r-project.org
