# Supplementary material for: Mycobacterial IHF is a highly dynamic nucleoid-associated protein that assists HupB in organizing chromatin
Source: Front Microbiol. 2023 Mar 7;14:1146406. doi: 10.3389/fmicb.2023.1146406 (PMC10028186; doi:10.3389/fmicb.2023.1146406)
Supplement: Supplementary file 1 [file Data_Sheet_1.PDF]

Text S1

Supplementary Experimental Procedures

M. smegmatis mc^2 155 mutant strains construction

The allelic replacement of the *msihf* gene (encoding msIHF, MSMEG_3050 protein) and *hupB* gene (encoding HupB, MSMEG_2389) respectively with *msihf-egfp*/ *msihf-mneongreen*/ *msihf-dendra2*/ *msihf-pamcherry*/ *msihf-halotag*, and *hupB-pamcherry*/ *hupB-Dendra2* was performed accordingly to Parish and Stoker (1). The *msihf* gene together with the flanking sequences was amplified on a template of *M. smegmatis* mc^2 155 (WT) chromosomal DNA and using primer pairs, IHF1_slic_Fw/IHF1_slic_Rv, IHF2_slic_Fw/ IHF2_slic_Rv and IHF1PAmCherry_slic_Fw/IHF1PAmCherry_slic_Rv, IHF2PAmCherry_slic_Fw/ IHF2PAmCherry_slic_Rv for *egfp* and *pamcherry* fusions, respectively. The *egfp* and *pamcherry* genes with a short linkers encoding 10 amino acid sequence at the 5′-terminus was PCR-amplified using primers EGFP_slic_Fw/EGFP_slic_Rv and PAmCherry_slic_Fw/PAmCherry_slic_Rv, respectively. PCR products were cloned into a p2NIL (kanR) vector using SLIC (Sequence and Ligation Independent Cloning), and the resulted plasmids were verified by sequencing. At the end, the pGoal17 cassette was cloned into the PacI site of each p2NIL derivative. In the case of *msihf-mneongreen*, *msihf-dendra2* and *msihf-halotag* fusion genes were amplified with a short linkers encoding 10-12 amino acid sequence at the 5′-terminus by PCR using primers mNeon_Hind_Fw/mNeon_Bam_Rv, dendra2_Fw/dendra2_Rv, and halotag_Fw/halotag_Rv, sequenced and subcloned into HindIII/BamHI site of p2NIL*msihf* derivative. For *hupB-pamcherry* and *hupB-dendra2* constructs, fusion genes were amplified with a short linkers encoding 10 amino acid sequence at the 5′-terminus by PCR using primers PAmCherry_BamHI_Fw/PAmCherry_KpnI_Rv and Dendra2_BamHI_Fw/Dendra2_BamHI_Rv, sequenced and subcloned into BamHI/KpnI site of p2NIL*hupB* derivative. Then the pGoal17 cassette was cloned into the PacI site of all p2NIL derivatives. *M. smegmatis* mc^2 155 and *M. smegmatis ΔhupB* (2) cells were transformed with 200 - 800 ng of NaOH/EDTA-treated plasmid DNA, and unmarked mutants were selected accordingly to procedure described previously (2, 3). DCO mutants were analyzed by PCR, Western blotting and/or DNA sequencing.

For construction of msIHF depletion strains the CRISPRi/dCas9 system was used (4). CRISPRi/dCas9 system from *Streptococcus theromphilus* allows to inhibit the transcription of the gene of interest. Cas9 protein deprived of its endonuclease activity (dCas9) is guided to the chosen chromosomal locus by the attached complementary RNA sequence (sgRNA, single guide RNA). After binding in the vicinity of the PAM (protospacer adjacent motif) sequence, dCas9 creates a spatial hindrance for the RNA polymerase. Expression of dCas9 is controlled by an inducible *tetR* promoter (induced with anhydrotetracycline, aTc). The chosen PAM sequence (5′-NNGGAAA-3′) located within the *msihf* gene enabled for 110.5-fold repression of the gene (4). sgRNA sequence was prepared by hybridization of oligo pair sgRNA_ihfMS_Fw/sgRNA_ihfMS_Rv and cloned to BsmBI site of pLJR962 (kan^R) vector. Obtained pLJR962 derivative was verified by sequencing. (using anhydrotetracycline, aTc as an inducer) *M. smegmatis* mc^2 155, *M. smegmatis msihf-egfp, M. smegmatis hupB-egfp* (2, 3)*, M. smegmatis dnaN-mcherry* (5) and *M. smegmatis parB-mcherry* (5, 6) strains were transformed with 15 ng of plasmid DNA, and mutants were selected using 50 µg/ml kanamycin. Induced msIHF depletion was verified by RT-qPCR and Western blotting. Additionally, to analyze the influence of dCas9 on *M. smegmatis* growth, *M. smegmatis msihf-egfp* strain was transformed with pLJR962 plasmid containing no sgRNA sequence (pLJR962Ø) creating a control strain. All oligonucleotides used for *M. smegmatis* mc^2 155 mutant strains construction are listed in Table S1. To determine the growth curves for *M. smegmatis* mc^2 155 mutant strains, cells were grown at 37˚C in a final volume of 300 µl 7H9

(supplemented with ADC and Tween80, and aTc if applicable), and optical density measurements were taken at 20 min intervals for 30 h using a Bioscreen C instrument.

RNA isolation

RNA was isolated with TriReagent (Invitrogen) as described previously (7). Approx. 40 ml of *M. smegmatis* culture was centrifuged (5,000×g for 5 min at 4°C), and then the cells were resuspended in 1 ml of TriReagent, and disrupted with BeatBeater (3 × 3min with 1 min intervals on ice). RNA was purified and treated with DNase I (RapidOut DNA Removal Kit, Invitrogen) according to the manufacturer's protocol. Obtained RNA quantity and purity were checked by agarose electrophoresis and using NanoDrop.

Reverse-Transcription and Quantitative PCR (RT-qPCR)

For cDNA synthesis up to 500 ng of RNA was used (Maxima First Strand cDNA synthesis kit; Thermo Fisher Scientific) in a final volume of 20 µl. The manufacturer protocol was modified for GC-rich *M. smegmatis* transcripts by increasing the synthesis temperature to 65°C. 10 ng of obtained cDNA was used for quantitative PCRs performed with PowerUp SYBR Green Master Mix (Applied Biosystems). The relative expression level of analyzed genes was quantified using the comparative ΔΔCt method, and the *sigA* gene was used as the endogenous control (StepOne Plus real-time PCR system, Applied Biosystems). The optimized oligonucleotides used in this study were synthetized by Sigma-Aldrich or Genomed (see Table S1).

1. Parish,T. and Stoker,N.G. (2000) Use of a flexible cassette method to generate a double unmarked Mycobacterium tuberculosis tlyA plcABC mutant by gene replacement. *Microbiol. Read. Engl.*, **146 (Pt 8)**, 1969–1975.

2. Hołówka,J., Trojanowski,D., Ginda,K., Wojtaś,B., Gielniewski,B., Jakimowicz,D. and Zakrzewska-Czerwińska,J. (2017) HupB Is a Bacterial Nucleoid-Associated Protein with an Indispensable Eukaryotic-Like Tail. *mBio*, **8**.

3. Hołówka,J., Trojanowski,D., Janczak,M., Jakimowicz,D. and Zakrzewska-Czerwińska,J. (2018) The Origin of Chromosomal Replication Is Asymmetrically Positioned on the Mycobacterial Nucleoid, and the Timing of Its Firing Depends on HupB. *J. Bacteriol.*, **200**.

4. Rock,J.M., Hopkins,F.F., Chavez,A., Diallo,M., Chase,M.R., Gerrick,E.R., Pritchard,J.R., Church,G.M., Rubin,E.J., Sassetti,C.M., *et al.* (2017) Programmable transcriptional repression in mycobacteria using an orthogonal CRISPR interference platform. *Nat. Microbiol.*, **2**, 16274.

5. Trojanowski,D., Ginda,K., Pióro,M., Hołówka,J., Skut,P., Jakimowicz,D. and Zakrzewska-Czerwińska,J. (2015) Choreography of the Mycobacterium replication machinery during the cell cycle. *mBio*, **6**, e02125-02114.

6. Ginda,K., Bezulska,M., Ziółkiewicz,M., Dziadek,J., Zakrzewska-Czerwińska,J. and Jakimowicz,D. (2013) ParA of Mycobacterium smegmatis co-ordinates chromosome segregation with the cell cycle and interacts with the polar growth determinant DivIVA. *Mol. Microbiol.*, **87**, 998–1012.

7. Kołodziej,M., Trojanowski,D., Bury,K., Hołówka,J., Paściak,M., Matysik,W., Kąkolewska,H., Feddersen,H., Giacomelli,G., Bramkamp,M., *et al.* (2020) Lsr2 is a nucleoid-associated protein that exerts pleiotropic effects on mycobacterial cellular processes. *bioRxiv*, 10.1101/2020.04.27.063487.
