# Supplementary material for: Mycobacterial IHF is a highly dynamic nucleoid-associated protein that assists HupB in organizing chromatin
Source: Front Microbiol. 2023 Mar 7;14:1146406. doi: 10.3389/fmicb.2023.1146406 (PMC10028186; doi:10.3389/fmicb.2023.1146406)
Supplement: Supplementary file 2 [file Image_1.PDF]

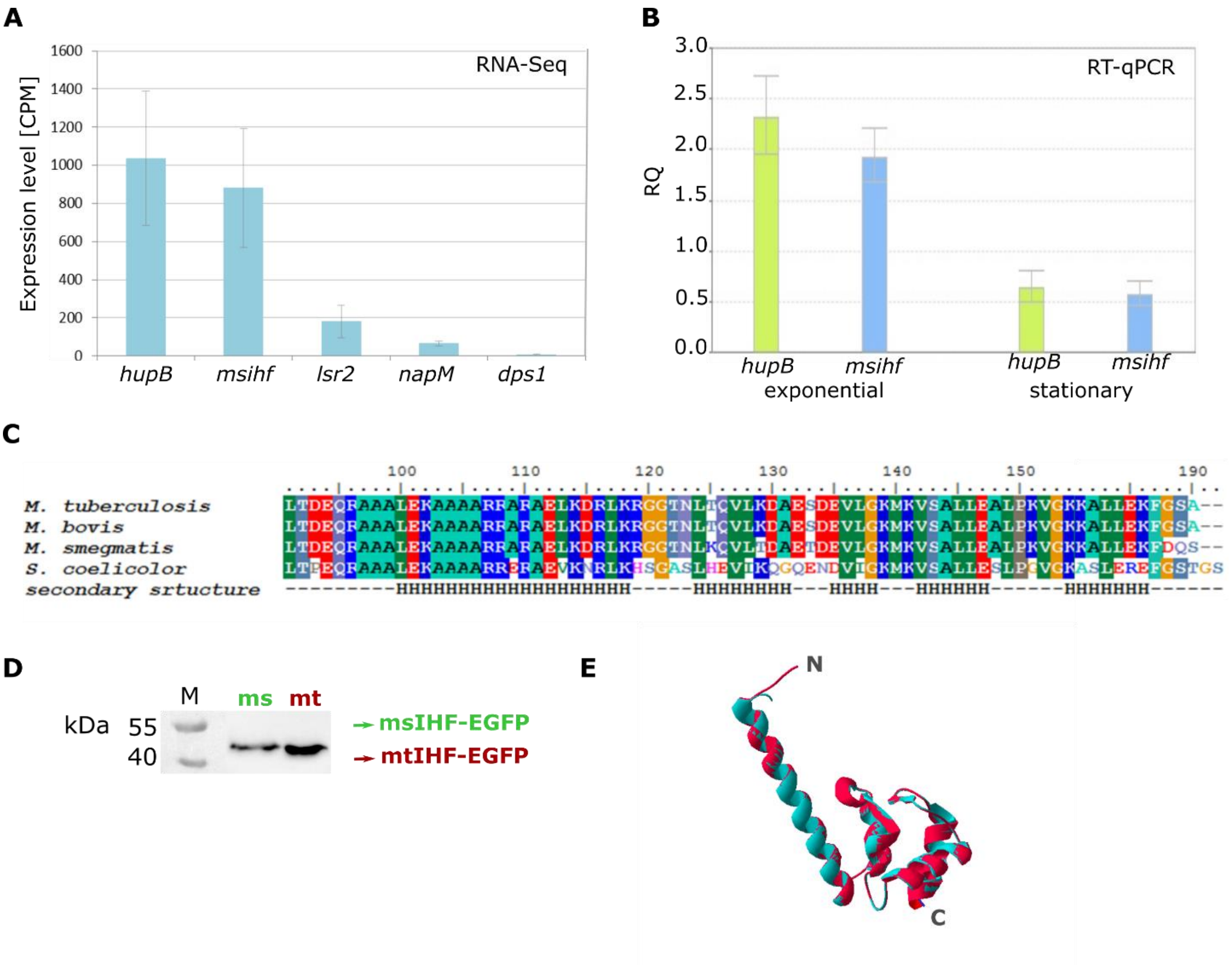

Fig. S1. Analysis of *msihf* gene expression level and the secondary structure of actinobacterial IHF proteins. A Expression levels of the main mycobacterial NAPs in the exponential phase of growth (RNA-Seq) (M. Kołodziej et al., mSphere https://doi.org/10.1128/mSphere.00290-21). CPM – counts per million. **B** The relative expression levels of *hupB* and *msihf* genes in exponential and stationary growth phases (RT-qPCR). RQ – relative quantification. **C** Multiple sequence alignment of mycobacterial IHF and sIHF (from *Streptomyces coelicolor*) proteins with secondary structure of mtIHF (H indicates α-helix structure). **D** Comparison of msIHF-EGFP and mtIHF-EGFP fusion proteins sizes was performed by Western blotting using mouse monoclonal anti-EGFP antibody (Invitrogen; dilution 1:1,000), followed by goat anti-rabbit IgG secondary antibody, conjugated with horseradish peroxidase (HRP, dilution 1:5,000; Invitrogen). Fusion proteins msIHF-EGFP and mtIHF-EGFP are marked in green and red, respectively. M – molecular weight marker. **E** A superimposed models of *M. smegmatis* (red) and *M. tuberculosis* (green) IHF proteins generated using Robetta protein structure prediction service (https://robetta.bakerlab.org/) and Swiss-PdbViewer.
