# Supplementary material for: Mycobacterial IHF is a highly dynamic nucleoid-associated protein that assists HupB in organizing chromatin
Source: Front Microbiol. 2023 Mar 7;14:1146406. doi: 10.3389/fmicb.2023.1146406 (PMC10028186; doi:10.3389/fmicb.2023.1146406)
Supplement: Supplementary file 3 [file Image_2.PDF]

A

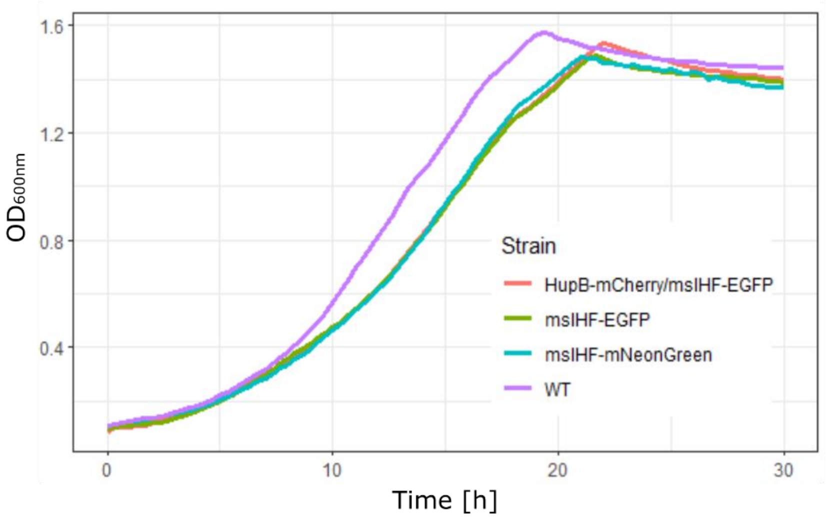

B

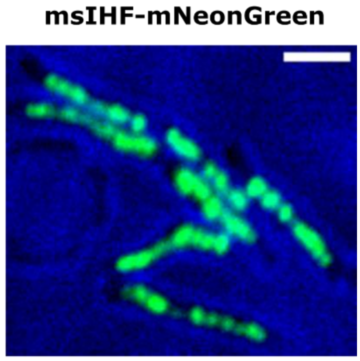

Fig. S2. Characterization of the constructed fluorescent reporter strains. A Growth curves of the constructed strains in comparison to *M. smegmatis* mc^2 155 wild type strain (WT). **B** Micrographs of representative cells of msIHF-mNeonGreen strain. Scale bar, 2 µm.
