# Supplementary material for: Mycobacterial IHF is a highly dynamic nucleoid-associated protein that assists HupB in organizing chromatin
Source: Front Microbiol. 2023 Mar 7;14:1146406. doi: 10.3389/fmicb.2023.1146406 (PMC10028186; doi:10.3389/fmicb.2023.1146406)
Supplement: Supplementary file 4 [file Image_3.PDF]

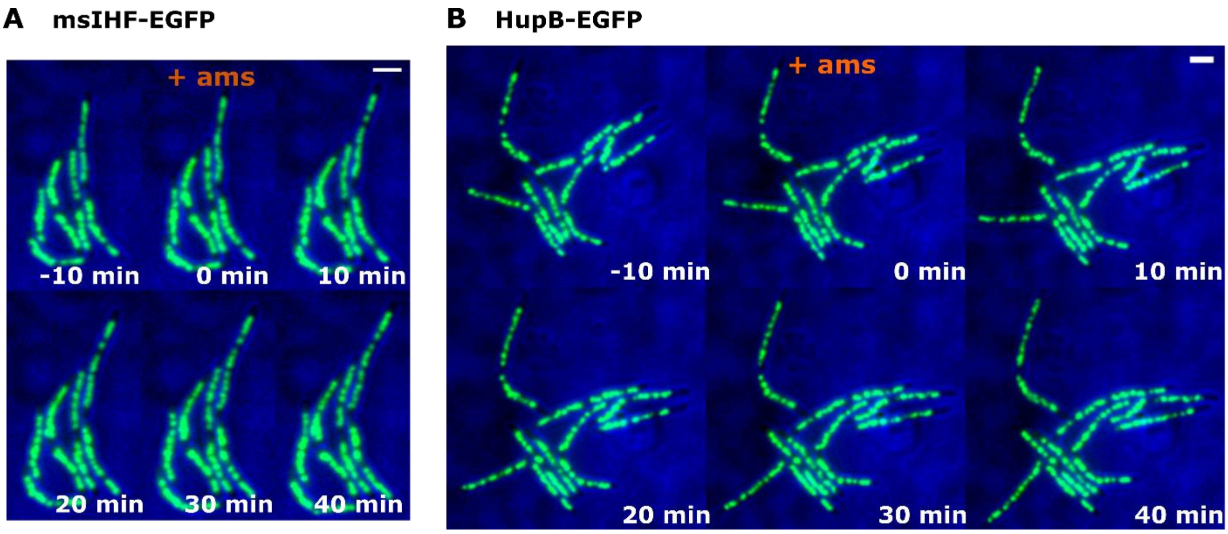

Fig. S3. msIHF-EGFP and HupB-EGFP cells upon amsacrine (ams) treatment. *M. smegmatis* cells of msIHF-EGFP **A** and HupB-EGFP **B** strains were exposed to amsacrine (final concentration, 31.5 µg/ml) for 6 h. Scale bar, 2 µm.
