# Supplementary material for: Mycobacterial IHF is a highly dynamic nucleoid-associated protein that assists HupB in organizing chromatin
Source: Front Microbiol. 2023 Mar 7;14:1146406. doi: 10.3389/fmicb.2023.1146406 (PMC10028186; doi:10.3389/fmicb.2023.1146406)
Supplement: Supplementary file 5 [file Image_4.PDF]

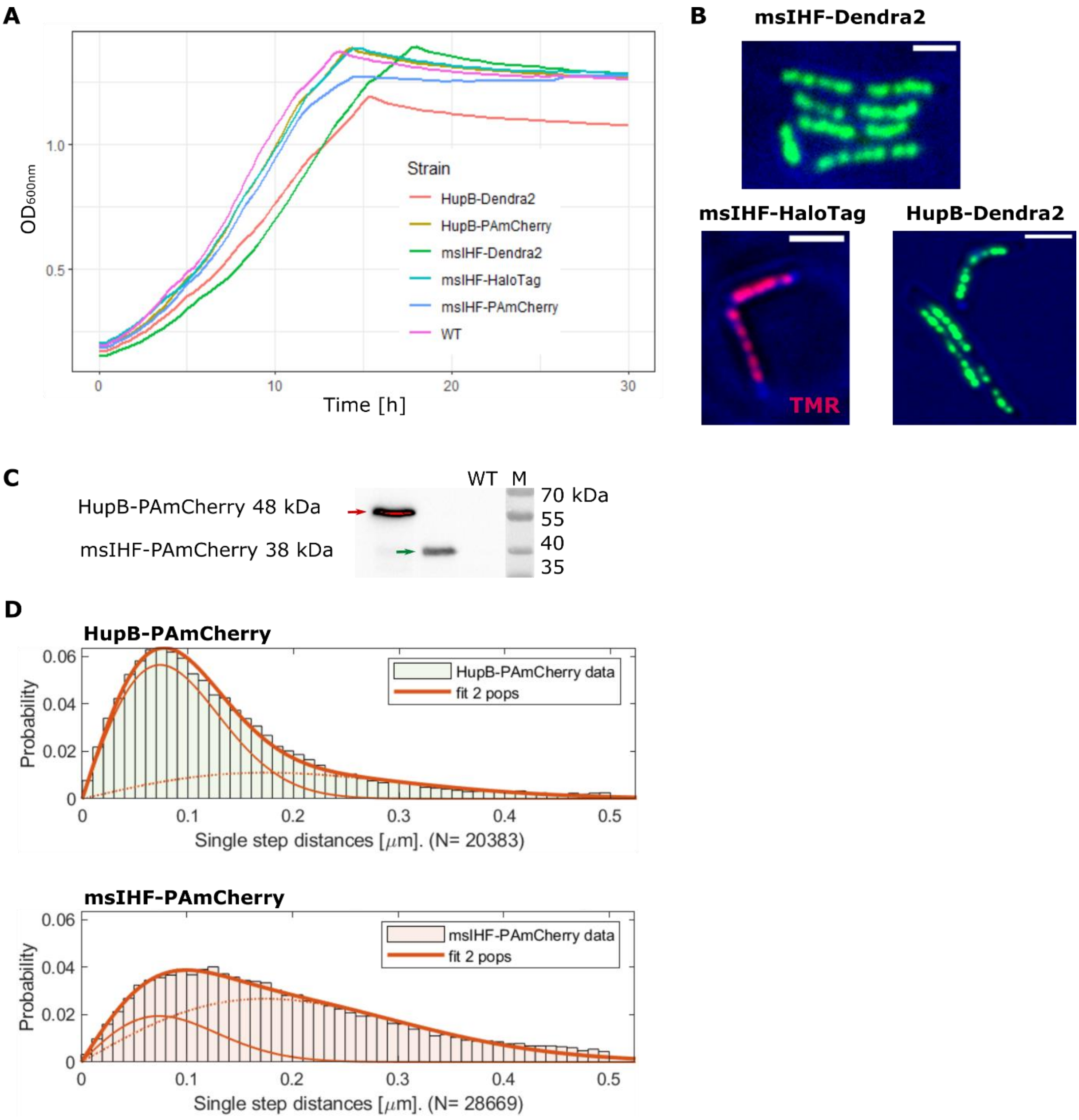


**Fig. S4. Characterization of the constructed fluorescent reporter strains used for SPT experiments. A** Growth curves of the analyzed strains in comparison to *M. smegmatis* mc$^2$ 155 wild-type strain (WT). **B** Micrographs of representative cells of msIHF-Dendra2, msIHF-HaloTag stained with 50 nM TMRdirect (Promega), and HupB-Dendra2 strains. Scale bar, 2 µm. **C** Production of fusion proteins of the expected sizes was confirmed by Western blotting using polyclonal anti-mCherry antibody produced in rabbit (Invitrogen; dilution 1:1,000), followed by goat anti-rabbit IgG secondary antibody, conjugated with horseradish peroxidase (HRP, dilution 1:5,000; Invitrogen). Green and red arrows indicate respectively msIHF-PAmCherry and HupB-PAmCherry; *M. smegmatis* mc$^2$ 155 wild-type strain (WT) served as a negative control. M – molecular weight marker. **D** Distribution of the frame-to-frame displacements (jump distances, JD) for HupB-PAmCherry and msIHF-PAmCherry with fast diffusive (dotted line) and confined (solid line) particles subpopulations determined by two-component model.
