# Supplementary material for: Mycobacterial IHF is a highly dynamic nucleoid-associated protein that assists HupB in organizing chromatin
Source: Front Microbiol. 2023 Mar 7;14:1146406. doi: 10.3389/fmicb.2023.1146406 (PMC10028186; doi:10.3389/fmicb.2023.1146406)
Supplement: Supplementary file 6 [file Image_5.PDF]

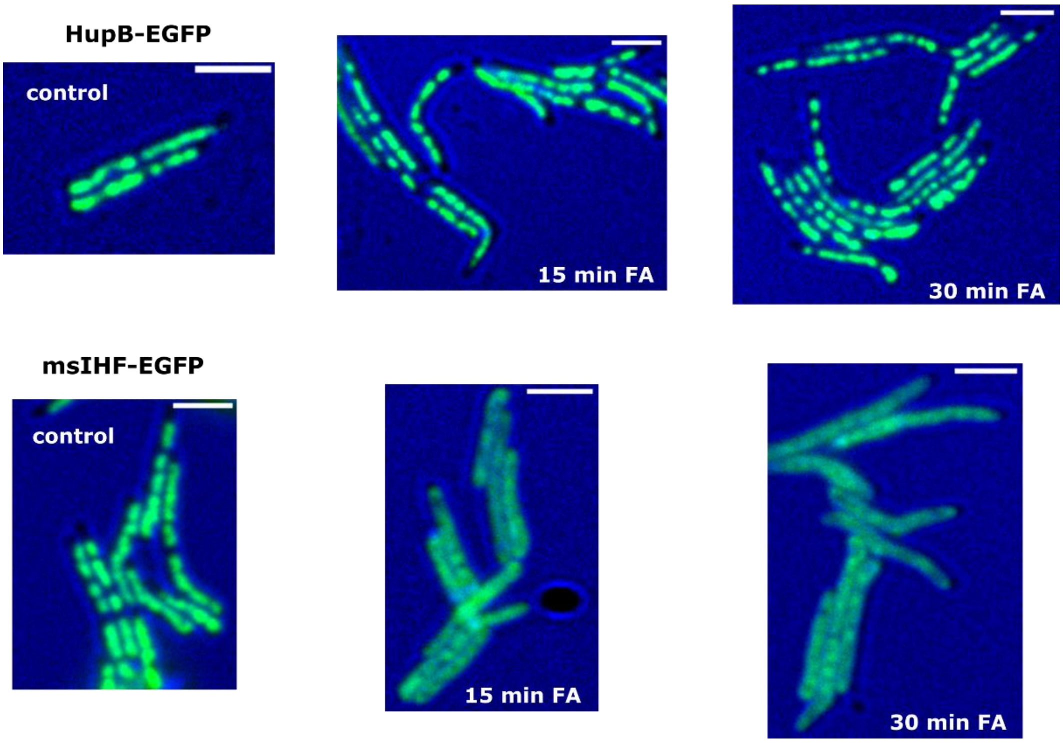

Fig. S5. Comparison of HupB-EGFP and msIHF-EGFP fluorescence patterns after formaldehyde fixation. Exponential phase cells were fixed with 1% formaldehyde for 15 or 30 min, then quenched with 160 mM glycine for 15 min, and smeared onto microscopic slides. Control – cells without fixation with formaldehyde, FA - formaldehyde. Scale bar, 2 µm.
