# Supplementary material for: Mycobacterial IHF is a highly dynamic nucleoid-associated protein that assists HupB in organizing chromatin
Source: Front Microbiol. 2023 Mar 7;14:1146406. doi: 10.3389/fmicb.2023.1146406 (PMC10028186; doi:10.3389/fmicb.2023.1146406)
Supplement: Supplementary file 7 [file Image_6.PDF]

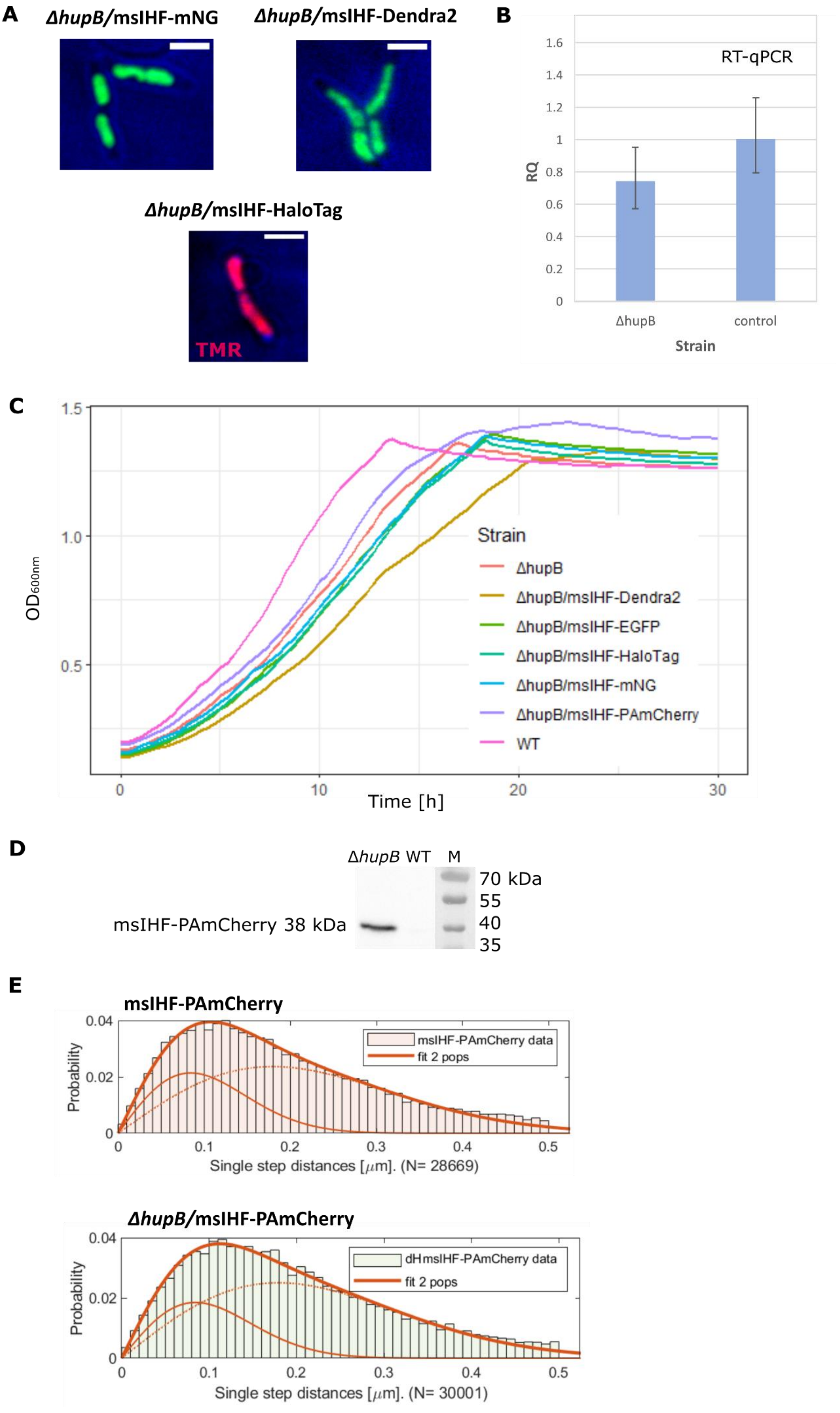

A
ΔhupB/msIHF-mNG
ΔhupB/msIHF-Dendra2
ΔhupB/msIHF-HaloTag
TMR
B
RT-qPCR
RQ
1.6
1.4
1.2
1
0.8
0.6
0.4
0.2
0
ΔhupB
control
Strain
C
1.5
1.0
0.5
OD600nm
0
10
20
30
Time [h]
Strain
ΔhupB
ΔhupB/msIHF-Dendra2
ΔhupB/msIHF-EGFP
ΔhupB/msIHF-HaloTag
ΔhupB/msIHF-mNG
ΔhupB/msIHF-PAmCherry
WT
D
ΔhupB WT M
70 kDa
55
40
35
msIHF-PAmCherry 38 kDa
E
msIHF-PAmCherry
Probability
0.04
0.02
0
0.1
0.2
0.3
0.4
0.5
msIHF-PAmCherry data
fit 2 pops
Single step distances [μm]. (N= 28669)
ΔhupB/msIHF-PAmCherry
dHmsIHF-PAmCherry data
fit 2 pops
Single step distances [μm]. (N= 30001)

Fig. S6. Characterization of the constructed fluorescent reporter strains in the Δ*hupB* genetic background. A Micrographs presenting the representative cells of the strains: Δ*hupB*/msIHF-mNeonGreen (Δ*hupB*/msIHF-mNG), *hupB*/msIHF-Dendra2, and Δ*hupB*/msIHF-HaloTag stained with 50 nM TMRdirect (Promega). Scale bar, 2 µm. **B** The relative expression level (RT-qPCR) of *msihf* gene in exponentially growing Δ*hupB* cells in comparison to the wild-type strain (control). RQ – relative quantification. **C** Growth curves of the analyzed strains in comparison to *M. smegmatis* mc^2 155 wild-type (WT) and Δ*hupB* strains. **D** Expression of fusion proteins of the expected sizes was confirmed by Western blotting using polyclonal anti-mCherry antibody produced in rabbit (Invitrogen; dilution 1:1,000), followed by goat anti-rabbit IgG secondary antibody, conjugated with horseradish peroxidase (HRP, dilution 1:5,000; Invitrogen). *M. smegmatis* mc^2 155 wild-type strain (WT) served as a negative control. M – molecular weight marker. **E** Distribution of the frame-to-frame displacements (jump distances, JD) for msIHF-PAmCherry in the wild type and Δ*hupB* genetic background with fast diffusive (dotted line) and confined (solid line) particles subpopulations determined by two-component model.
