# Supplementary material for: Mycobacterial IHF is a highly dynamic nucleoid-associated protein that assists HupB in organizing chromatin
Source: Front Microbiol. 2023 Mar 7;14:1146406. doi: 10.3389/fmicb.2023.1146406 (PMC10028186; doi:10.3389/fmicb.2023.1146406)
Supplement: Supplementary file 8 [file Image_7.PDF]

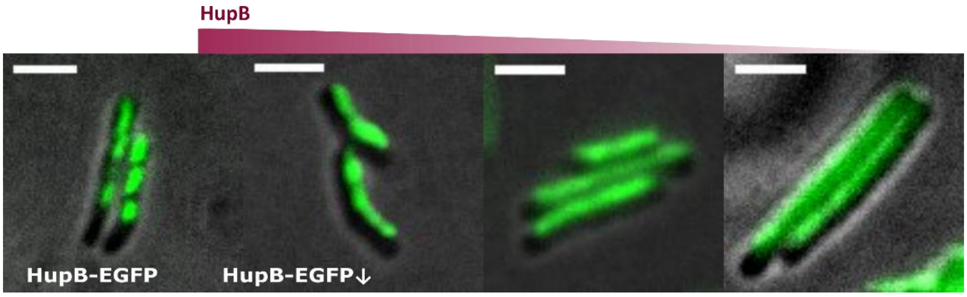

Fig. S7 Changes in the HupB-EGFP fluorescence pattern under decreasing HupB level (HupB-EGFP↓ strain). The *hupB-egfp* fusion gene under inducible promoter was introduced in Δ*hupB* strain (J. Hołówka et al., mBio https://doi.org/10.1128/mBio.01272-17), decreasing concentration of inducer (i.e., 0.1, 0.05, 0.01% of acetamide, respectively) was used to deplete HupB-EGFP level. Scale bar, 2 µm.
