# Supplementary material for: Mycobacterial IHF is a highly dynamic nucleoid-associated protein that assists HupB in organizing chromatin
Source: Front Microbiol. 2023 Mar 7;14:1146406. doi: 10.3389/fmicb.2023.1146406 (PMC10028186; doi:10.3389/fmicb.2023.1146406)
Supplement: Supplementary file 9 [file Image_8.PDF]

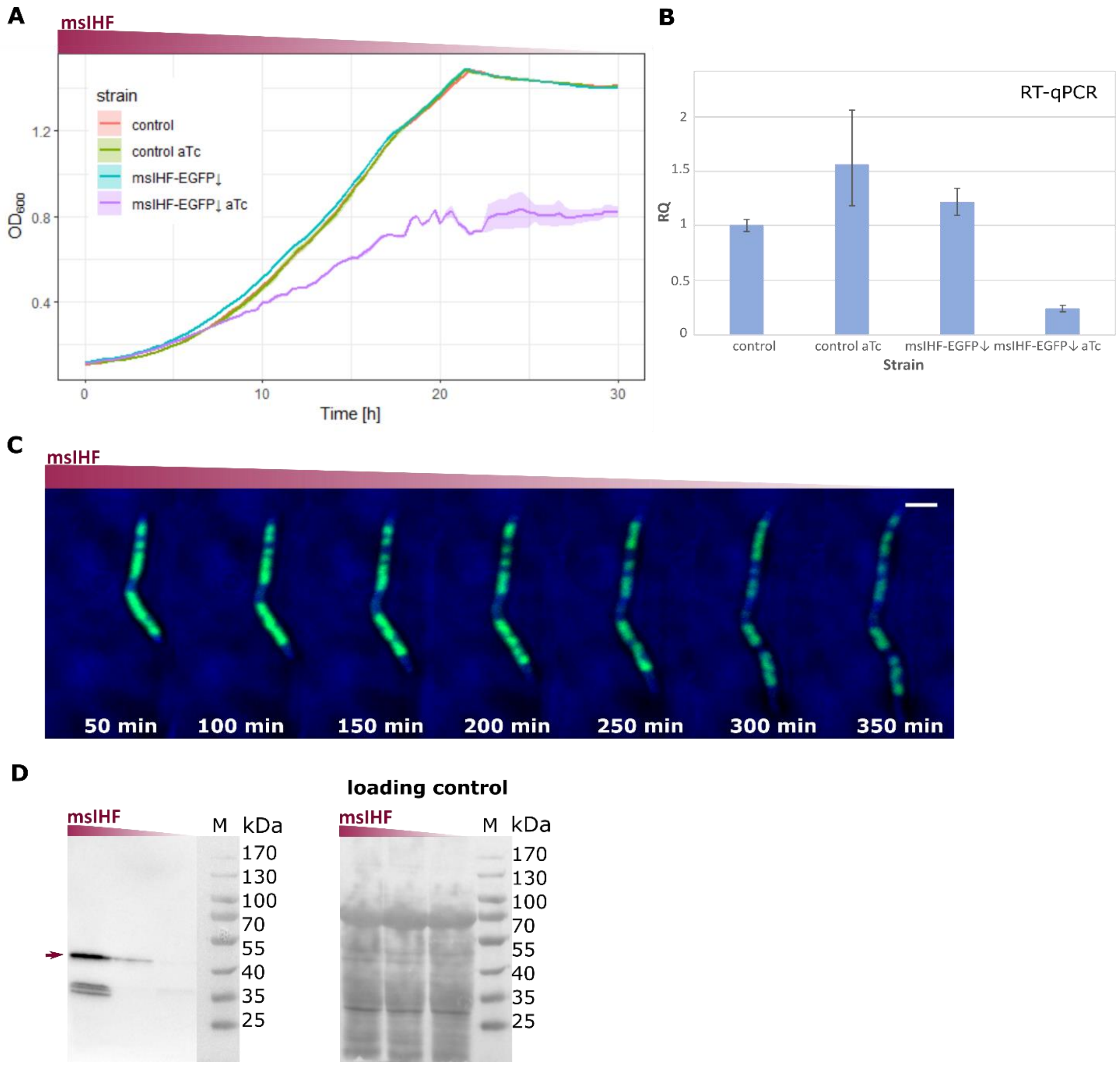

Fig. S8. Characterization of the msIHF-EGFP↓ strain. A Growth curves of msIHF-EGFP↓ and control strain (carrying no sgRNA sequence; to verify effect of dCas9 on growth of msIHF-EGFP strain upon induction) without the inducer and upon 50 ng/ml aTc induction. **B** The relative expression level of *msihf* gene in msIHF-EGFP↓ in comparison to the control strain without induction and after 6 h of induction with 50 ng/ml aTc (RT-qPCR). RQ – relative quantification. **C** Time-lapse experiment showing decreasing level of msIHF-EGFP upon 50 ng/ml aTc induction (n=100). Scale bar, 2 µm. **D** Western blotting analysis of msIHF-EGFP fusion protein level in msIHF-EGFP↓ strain without induction, after approx. 6 h and 24 h of induction with 50 ng/ml aTc. Western blotting was performed using anti-EGFP antibody (Invitrogen; dilution 1:1,000), followed by goat anti-mouse IgG secondary antibody, conjugated with horseradish peroxidase (HRP, dilution 1:5,000; Invitrogen); violet arrow indicates msIHF-EGFP fusion protein (38 kDa). Loading control was conducted utilizing MemCode Reversible Protein Stain Kit (Thermo Scientific).
