# Supplementary material for: Mycobacterial IHF is a highly dynamic nucleoid-associated protein that assists HupB in organizing chromatin
Source: Front Microbiol. 2023 Mar 7;14:1146406. doi: 10.3389/fmicb.2023.1146406 (PMC10028186; doi:10.3389/fmicb.2023.1146406)
Supplement: Supplementary file 10 [file Image_9.PDF]

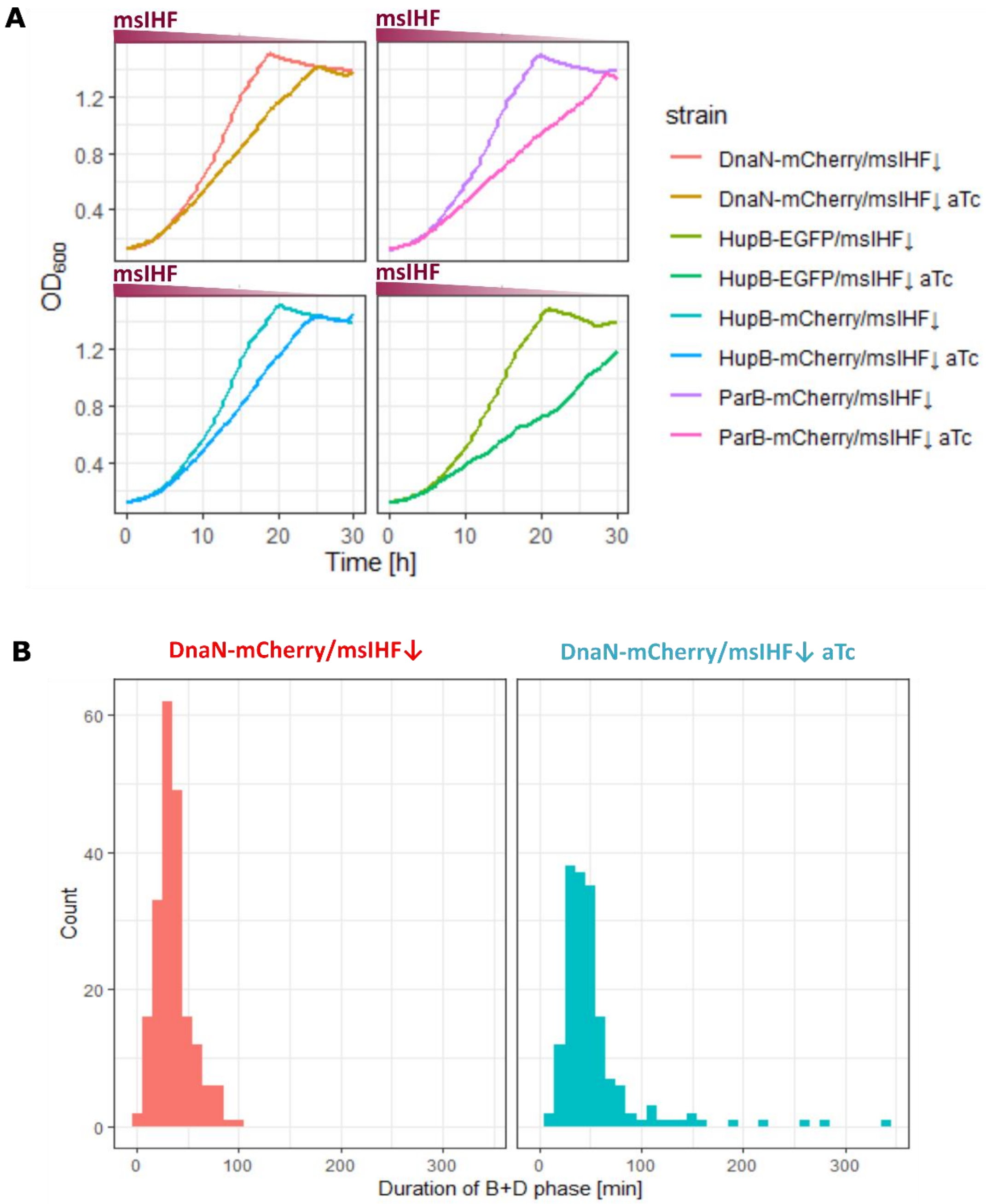

Fig. S9. Influence of decreasing msIHF level on the replication dynamics. A Growth curves of HupB-EGFP/msIHF↓, HupB-mCherry/msIHF↓, DnaN-mCherry/msIHF↓, ParB-mCherry/msIHF↓ without the inducer and upon 50 ng/ml aTc induction. **B** Histograms presenting distribution of B+D phase duration time in DnaN-mCherry/msIHF↓ strain without the inducer (red) and upon 50 ng/ml aTc induction (blue).
