# Supplementary material for: Mycobacterial IHF is a highly dynamic nucleoid-associated protein that assists HupB in organizing chromatin
Source: Front Microbiol. 2023 Mar 7;14:1146406. doi: 10.3389/fmicb.2023.1146406 (PMC10028186; doi:10.3389/fmicb.2023.1146406)
Supplement: Supplementary file 11 [file Image_10.PDF]

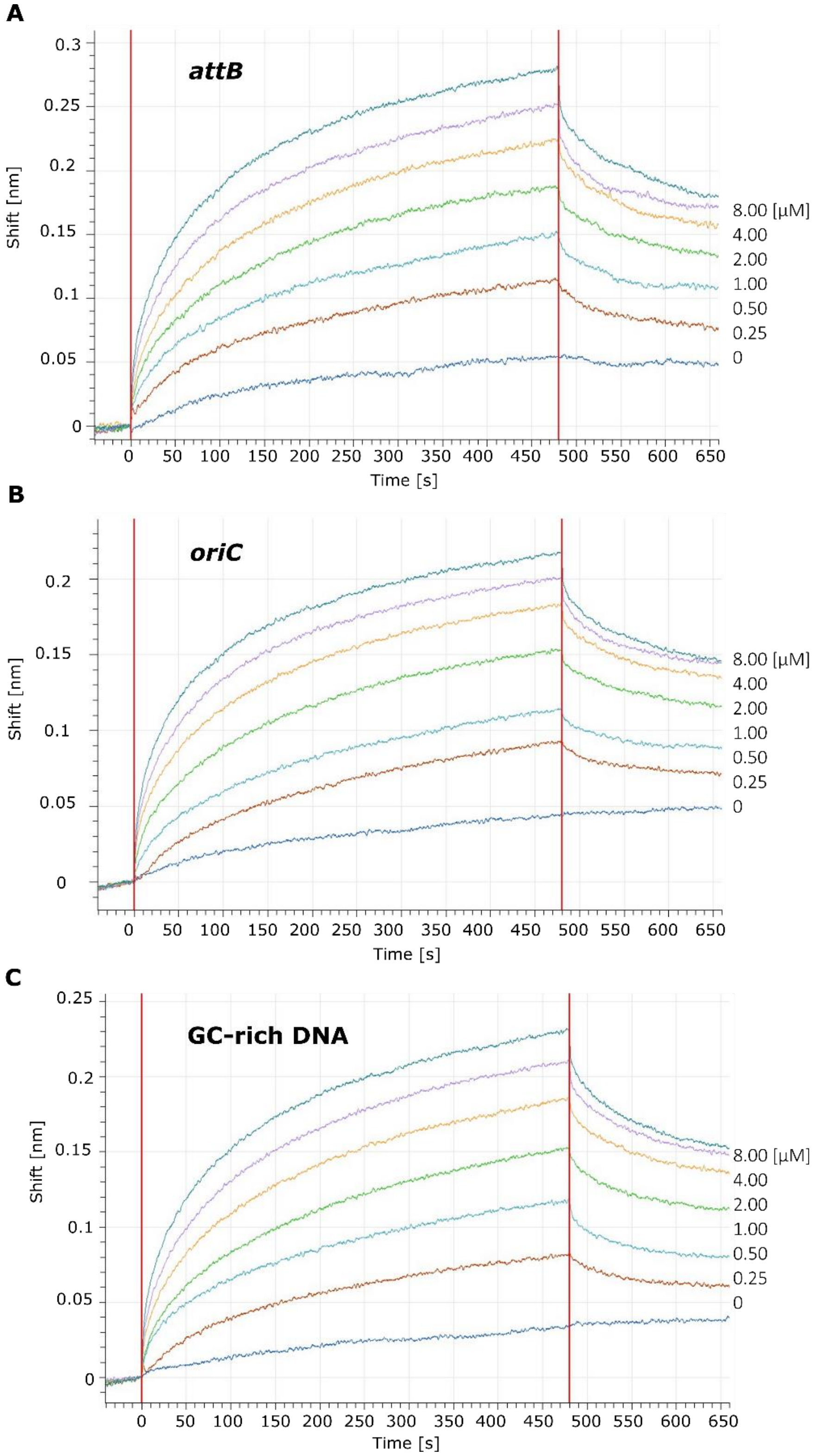

Fig. S10. Analysis of msIHF DNA binding *in vitro*. BLI sensogram presents the binding of increasing concentrations of msIHF (0, 0.25, 0.50, 1.00, 2.00, 4.00, and 8.00 µM) to the *attB* site (**A**), *oriC* region (**B**), and GC-rich DNA (**C**), as prepared using a ForteBio Octet K2 system and Streptavidin biosensors (SAX2) (Pall ForteBio).
