# Supplementary material for: Mycobacterial IHF is a highly dynamic nucleoid-associated protein that assists HupB in organizing chromatin
Source: Front Microbiol. 2023 Mar 7;14:1146406. doi: 10.3389/fmicb.2023.1146406 (PMC10028186; doi:10.3389/fmicb.2023.1146406)
Supplement: Supplementary file 12 [file Table_1.pdf]

Table S1. Oligonucleotides used during the study.

Primer	5' – 3' sequence	Application
IHF1_slic_Fw	TAACTGTGATAAACTACCGCATTAAAGCTTTCAAGGCCGTGATGTGCAT	Construction of *M. smegmatis* mc^2 mutant strains (see Text S1).
IHF1_slic_Rv	CCGGGCCCGGCAGCGACTGGTCGAACTTCTCCAG	
IHF2_slic_Fw	CTGTACAAGTAAGGCGCGCAGATTGACTG	
EGFP_slic_Fw	TTCGACCAGTCGCTGCCGGGCCCGGAGC	
EGFP_slic_Rv	CAATCTGCGCGCCTTACTTGTACAGCTCGTCCATGCC	
IHF1PAmCherry_slic_Fw	TAACTGTGATAAACTACCGCATTAACCGGTCACGCCTGGT	
IHF1PAmCherry_slic_Rv	CAGCCGAAAGCTTCGACTGGTCGAACTTCTCCAG	
IHF2PAmCherry_slic_Fw	AAGTAAGGATCCGGCGCGCAGATTGACTGT	
IHF2PAmCherry_slic_Rv	TTAGGTGACACTATAGAATACATAGCATCGGCGTGCGGG	
PAmCherry_slic_Fw	TTCGACCAGTCGAAGCTTTCGGCTGGCTCC	
PAmCherry_slic_Rv	CAATCTGCGCGCCGGATCCTTACTTGTACAGCTCGTCC	
mNeon_Hind_Fw	CCAAGCTTATGTCGGCTGGCTCCGCTGCTGG	
mNeon_Bam_Rv	CCGGATCCTTATTTGTACAATTCATCCATG	
dendra2_Fw	CCAAGCTTTCGGCTGGCTCCGCTGCTGGTTCTGGCGAATTCAACACCCCGGGAATTAACC	
dendra2_Rv	CCGGATCCTTACACACCTGGCTGGGCAGGGGGC	
halotag_Fw	AAGCTTTCGGCTGGCTCCGCTGCTGGTTCTGGCGAATTCATGGCAGAAATCGGTACTGG	
halotag_Rv	GGATCCTTAGCCGGAAATCTCGAGCGTCGAC	
PAmCherry_BamHI_Fw	CCGGATCCTCGGCTGGCTCCGCTGCTGG	
PAmCherry_KpnI_Rv	CCGGTACCTTACTTGTACAGCTCGTCCATGC	
Dendra2_BamHI_Fw	CCGGATCCTCGGCTGGCTCCGCTGCTGGTTCTGGCGAATTCAACACCCCGGGAATTAACC	
Dendra2_KpnI_Rv	CCGGTACCTTACACACCTGGCTGGGCAGGGGGC	
sgRNA_ihfMS_Fw	AAACGCTGCTGGAAGCTCTGCCCAAGGT	Construction of *M. smegmatis* mc^2 msIHF depletion strains (Text S1).
sgRNA_ihfMS_Rv	GGGAACCTTGGGCAGAGCTTCCAGCAGC	
ms_attB_FW	TTTGAACCTGCGGCCTTCCGCT	Amplification DNA fragments for EMSA experiments.
ms_attB_RV	GCAACGAAAAAGGGACCGCACGT	
GC_msmeg_FW	GTGCCGAAGGCTCCCACGGC	
GC_msmeg_RV	CTGCTGTGCTGTTCGGCTGCACGTA	
ms_oriC_FW	GCTCGGAGACTCAGCCCAC	

ms_oriC_RV	TTGCCCAGCATCCTCGGTG	
**Primer**	**5’ – 3’ sequence**	**Application**
M13 Fwd	GTAAAACGACGGCCAGT	Amplification of biotin- or IR700 labelled DNA fragments for BLI and EMSA experiments.
SP6 Rv BTN	Biotin-CAGGAAACAGCTATGAC	
SP6 Rv IR700	IR700- CAGGAAACAGCTATGAC	
P-1	AAGGCGCCGGATCCGAATTCGTGGCCCTTCCCCAGTTG	Amplification of *msihf* gene for msIHF protein purification.
P-2	TCGAGTGCGGCCGCAAGCTTTTACGACTGGTCGAACTTCTCCAG	
RT_MsIHF_Fw	GTGGCCCTTCCCCAGTTG	Primers utilized for RT-qPCR experiments (Text S1).
RT_MsIHF_Rv	CGCTTGAGTCGATCCTTGAG	
RT_MshupB_Fw	TGAACAAAGCGGAGCTCATC	
RT_MshupB_Rv	CGCACGATGGTGTCGACGA	
RT_MssigA_Fw	GCGGCATGGCGTTCCT	
RT_MssigA_Rv	CGAGAACTTGTAGCCCTTGGTGTA	
