# Supplementary material for: Mycobacterial IHF is a highly dynamic nucleoid-associated protein that assists HupB in organizing chromatin
Source: Front Microbiol. 2023 Mar 7;14:1146406. doi: 10.3389/fmicb.2023.1146406 (PMC10028186; doi:10.3389/fmicb.2023.1146406)
Supplement: Supplementary file 13 [file Table_2.pdf]

Table S2. Average time of daughter chromosomes separation in the Δ*hupB* mutant strain (Δ*hupB*/msIHF-EGFP) in comparison to the wild-type strain (msIHF-EGFP).

Strain	Transmittance [%], exposure time [min]			
	**32%, 35 ms**	**32%, 50 ms**	**32%, 80 ms**	**50%, 80 ms**
Δ*hupB*/msIHF-EGFP	167 min	162 min	196 min	193 min
msIHF-EGFP	151 min	152 min	167 min	167 min
